# Cognitive Behavioral Therapy for Veterans With Comorbid Posttraumatic Headache and Posttraumatic Stress Disorder Symptoms: A Randomized Clinical Trial

**DOI:** 10.1001/jamaneurol.2022.1567

**Published:** 2022-06-27

**Authors:** Donald D. McGeary, Patricia A. Resick, Donald B. Penzien, Cindy A. McGeary, Timothy T. Houle, Blessen C. Eapen, Carlos A. Jaramillo, Paul S. Nabity, David E. Reed, John C. Moring, Lindsay M. Bira, Hunter R. Hansen, Stacey Young-McCaughan, Briana A. Cobos, Jim Mintz, Terence M. Keane, Alan L. Peterson

**Affiliations:** 1 Department of Psychiatry and Behavioral Sciences, The University of Texas Health Science Center San Antonio, San Antonio; 2 Department of Rehabilitation Medicine, The University of Texas Health Science Center at San Antonio, San Antonio; 3 South Texas Veterans Health Care System, San Antonio; 4 Department of Psychology, The University of Texas at San Antonio, San Antonio; 5 Department of Psychiatry and Behavioral Sciences, Duke Health, Durham, North Carolina; 6 Departments of Psychiatry and Behavioral Medicine & Neurology, Wake Forest University, Winston-Salem, North Carolina; 7 Department of Anesthesia, Massachusetts General Hospital, Boston; 8 Greater Los Angeles Veterans Health Care System, Los Angeles, California; 9 Department of Physical Medicine and Rehabilitation, David Geffen School of Medicine, University of California, Los Angeles, Los Angeles; 10 Behavioral Science Division, National Center for PTSD, Boston, Massachusetts; 11 VA Boston Healthcare System, Boston, Massachusetts; 12 Department of Psychiatry, Boston University School of Medicine, Boston, Massachusetts

## Abstract

**Importance:**

Posttraumatic headache is the most disabling complication of mild traumatic brain injury. Posttraumatic stress disorder (PTSD) symptoms are often comorbid with posttraumatic headache, and there are no established treatments for this comorbidity.

**Objective:**

To compare cognitive behavioral therapies (CBTs) for headache and PTSD with treatment per usual (TPU) for posttraumatic headache attributable to mild traumatic brain injury.

**Design, Setting, and Participants:**

This was a single-site, 3–parallel group, randomized clinical trial with outcomes at posttreatment, 3-month follow-up, and 6-month follow-up. Participants were enrolled from May 1, 2015, through May 30, 2019; data collection ended on October 10, 2019. Post-9/11 US combat veterans from multiple trauma centers were included in the study. Veterans had comorbid posttraumatic headache and PTSD symptoms. Data were analyzed from January 20, 2020, to February 2, 2022.

**Interventions:**

Patients were randomly assigned to 8 sessions of CBT for headache, 12 sessions of cognitive processing therapy for PTSD, or treatment per usual for headache.

**Main Outcomes and Measures:**

Co–primary outcomes were headache-related disability on the 6-Item Headache Impact Test (HIT-6) and PTSD symptom severity on the PTSD Checklist for *Diagnostic and Statistical Manual of Mental Disorders* (Fifth Edition) (PCL-5) assessed from treatment completion to 6 months posttreatment.

**Results:**

A total of 193 post-9/11 combat veterans (mean [SD] age, 39.7 [8.4] years; 167 male veterans [87%]) were included in the study and reported severe baseline headache-related disability (mean [SD] HIT-6 score, 65.8 [5.6] points) and severe PTSD symptoms (mean [SD] PCL-5 score, 48.4 [14.2] points). For the HIT-6, compared with usual care, patients receiving CBT for headache reported −3.4 (95% CI, −5.4 to −1.4; *P* < .01) points lower, and patients receiving cognitive processing therapy reported −1.4 (95% CI, −3.7 to 0.8; *P* = .21) points lower across aggregated posttreatment measurements. For the PCL-5, compared with usual care, patients receiving CBT for headache reported −6.5 (95% CI, −12.7 to −0.3; *P* = .04) points lower, and patients receiving cognitive processing therapy reported −8.9 (95% CI, −15.9 to −1.9; *P* = .01) points lower across aggregated posttreatment measurements. Adverse events were minimal and similar across treatment groups.

**Conclusions and Relevance:**

This randomized clinical trial demonstrated that CBT for headache was efficacious for disability associated with posttraumatic headache in veterans and provided clinically significant improvement in PTSD symptom severity. Cognitive processing therapy was efficacious for PTSD symptoms but not for headache disability.

**Trial Registration:**

ClinicalTrials.gov Identifier: NCT02419131

## Key Points

**Question:**

Do cognitive behavioral therapies for posttraumatic headache and posttraumatic stress disorder (PTSD) symptoms improve headache-related disability in veterans compared with treatment per usual?

**Findings:**

A randomized clinical trial of 193 post-9/11 combat veterans with posttraumatic headache and PTSD symptoms found headache disability was significantly improved with cognitive behavioral therapy for headaches compared with treatment per usual. Though participants randomly assigned to cognitive processing therapy reported significantly greater improvement in PTSD symptom severity compared with treatment per usual, there was no significant effect of cognitive processing therapy on headache disability.

**Meaning:**

Cognitive behavioral therapies are efficacious treatments for veterans with comorbid posttraumatic headache and PTSD symptoms.

## Teaser

This randomized clinical trial investigates if cognitive behavioral therapies for posttraumatic stress disorder (PTSD) and posttraumatic headache improve headache-related disability and PTSD symptom severity in US veterans compared with treatment per usual.

## Introduction

Military service members and veterans are at high risk for head injury compared with civilians.^1,2^ More than 369 000 US veterans have at least 1 mild TBI (mTBI),^3^ and many will report onset or worsening of headache (ie, posttraumatic headache; PTH) within 3 months of their head injury (though the connection between headache and head injury is sometimes unclear).^4,5^ Veterans are more likely than civilians to develop PTH after mTBI,^1,6,7^ and mechanisms of PTH are poorly understood.^1,8^ Clinical posttraumatic stress disorder (PTSD) symptoms are common among veterans with mTBI,^5,9,10^ and PTSD comorbid with mTBI may increase risk for PTH onset, severity, and disability^11,12^ while diminishing treatment response.^9^

There are no confirmed frontline treatments for PTH attributable to mTBI. Ongoing research offers some support for neuromodulation and pharmacotherapy for mTBI-related headache,^11^ but at least 1 preventive pharmacotherapy will fail in 79% of those with headache after mTBI,^13^ and existing pharmacotherapy studies are low quality.^14^ Manualized cognitive behavioral therapies (CBTs) offer safe, broad-spectrum treatment for PTH,^2,15,16^ but limited evidence supports their use.^17,18^ Indeed, the only published trial of behavioral treatment for PTH found no benefit in a civilian sample.^19^ Veterans with PTH may respond to treatment differently than civilians owing to a higher risk of comorbid PTSD symptoms,^20^ but at the time of this article, there are no published randomized clinical trials in veterans for any PTH intervention.

The present study examined 2 nonpharmacological interventions for PTH in military veterans with persistent headache related to comorbid mTBI and clinical PTSD symptoms. Investigators compared a manualized behavioral headache intervention, cognitive behavioral therapy for headache (CBTH), and a manualized PTSD intervention, cognitive processing therapy (CPT),^21^ with usual care in a sample of US military veterans with mTBI-related headache. CBTH uses cognitive behavioral therapy concepts with documented effects for headache disability and comorbid mood in primary headaches,^22^ and CPT has produced significant improvements in PTSD and related health symptoms (including headaches).^23^ We hypothesized that both CBTH and CPT would result in significant improvement in both headache-related disability and PTSD symptom severity compared with usual care in veterans with PTH attributable to mTBI and comorbid clinical PTSD symptoms.

## Methods

### Ethical Considerations

This clinical trial was approved through a joint university and Department of Veterans Affairs (VA) institutional review board and Duke Health institutional review board and monitored by the US Army Medical Research and Development Command Human Research Protection Office. Participants provided written informed consent and were eligible for financial compensation. The trial research protocol was previously published (Supplement 1 and Supplement 2).^24^ This study followed the Consolidated Standards of Reporting Trials (CONSORT) reporting guidelines.

### Study Population

**Figure 1.**
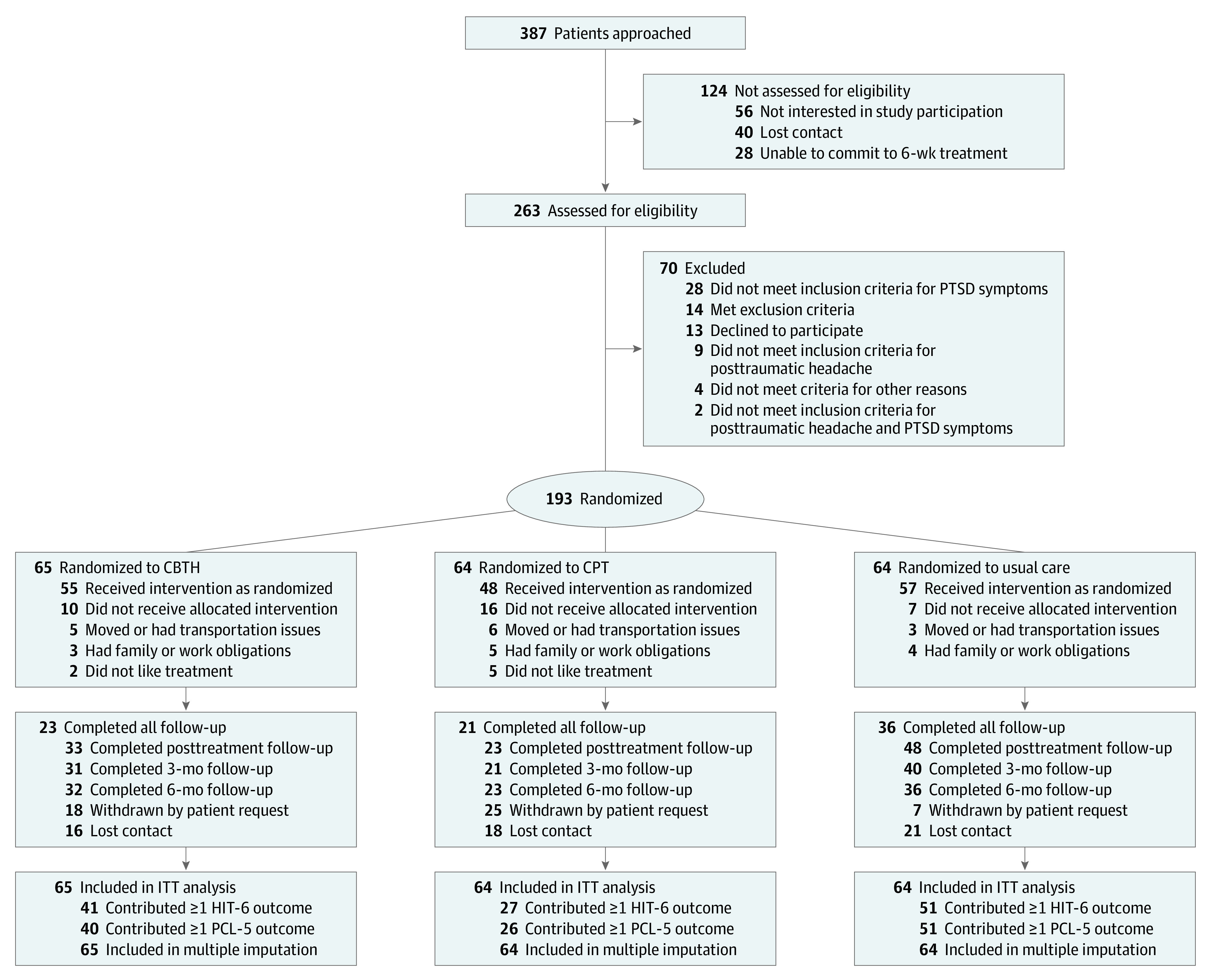
Consolidated Standards of Reporting Trials (CONSORT) Flow Diagram CBTH indicates cognitive behavioral therapy for headache; CPT, cognitive processing therapy; HIT-6, 6-Item Headache Impact Test; ITT, intention to treat; PCL-5, PTSD Checklist for *DSM-5*; PTSD; posttraumatic stress disorder.

Trial participants were recruited through a VA hospital, military facilities, and the community. Race and ethnicity were assessed by self-report on a standardized demographics questionnaire. Race categories included American Indian, Asian, Black/African American, Native Hawaiian, White, and other (a free-text field for identification of race was not included on this list). Ethnicity categories included Hispanic and non-Hispanic. Assessment of race and ethnicity was required by the funder. Figure 1 details who was approached for screening, including veterans, active-duty military, National Guard members, and Reservists with at least 1 deployment after September 11, 2001. All met International Classification of Headache Disorders (ICHD) criteria for persistent (ICHD-3, 5.2.2) or delayed-onset (ICHD-3, A5.2.2.1) headache attributable to mTBI (ie, posttraumatic headache) based on a structured diagnostic interview for headache and confirmed by a VA physician (B.C.E., C.A.J.) with expertise in headache. Qualifying participants had confirmed mTBI based on established case definition,^25^ stable headache medication dosage (ie, no change during study participation), and clinically significant PTSD symptoms (baseline *Diagnostic and Statistical Manual of Mental Disorders* [Fifth Edition] [*DSM-5*] [PCL-5] score of 25 or more and exposure to a traumatic event, 1 or more intrusion symptoms, and 1 or more avoidance symptoms based on the Clinician-Administered PTSD Scale for *DSM-5*).^26^

Individuals were excluded if they experienced significant change in headache symptoms within 6 weeks of enrollment, recently or currently engaged in active PTSD treatment, were diagnosed with medication overuse headache, required immediate psychiatric or medical intervention, or demonstrated cognitive impairment affecting ability to participate.

### Study Design

This 3-group randomized clinical trial compared CBTH and CPT with treatment per usual (TPU) at a large VA multiple-trauma rehabilitation center. Participants were randomized in a 1:1:1 ratio using computerized, central randomization and blind allocation. The standardized treatment window for the study was 6 weeks, although patients received additional time to complete treatment if they missed sessions.^24^ Participants were asked to complete assessments at pretreatment, posttreatment, 3-month, and 6-month posttreatment follow-ups (Figure 1).

### Study Treatments

CBTH was delivered in 8 weekly or biweekly, 1-hour sessions by trained, licensed clinical psychologists (P.S.N., J.C.M.) or clinical psychology postdoctoral fellows (L.M.B., H.R.H.). Treatment professionals completed a 2-hour orientation to the manual and demonstrated competency with 2 supervised clinical cases.^24^ CBTH professionals received expert clinical supervision from study investigators.

CPT is a manualized intervention for PTSD delivered in 12-hour sessions over 6 weeks.^21,23^ All study CPT professionals completed standardized training on PTSD and CPT treatment, regular supervision and case consultation, and a competency process consistent with best practices for CPT training.^27^

TPU was consistent with multidisciplinary treatment in a large VA multiple-trauma center, allowing for comparison between experimental interventions for PTH (CBTH and CPT) and the highest standard of VA clinical care for mTBI.^28,29,30,31^ Participants who received TPU received it in the form of pharmacotherapies, interventional pain management (eg, Botox injection), physical therapy, and complementary and integrative health treatments (eg, massage, acupuncture).

### Behavioral Treatment Overlap

CBTH and CPT were both developed using a CBT framework. The CBTH intervention focused exclusively on headache and stress, relying heavily on behavioral interventions and stress management with some cognitive therapy. CPT focused exclusively on PTSD, emphasizing cognitive therapy (eTable 1 in Supplement 3).

### Efficacy Assessments and Outcomes

Study participants completed a standardized assessment battery administered by blinded assessors at pretreatment, posttreatment, 3-month, and 6-month follow-up. A detailed description of blinding, allocation, measures, and timelines is included in a published methods article.^24^ The primary headache end point for this trial was the 6-Item Headache Impact Test (HIT-6),^32^ a self-reported disability measure assessing changes in quality of life and functioning related to chronic headache.^33,34^ The HIT-6 has been used as the primary end point in other PTH studies^12^ and is recommended as an outcome in guidelines for trials of behavioral treatment in headache.^35^ PTSD symptoms were assessed using the PCL-5, a 20-item, self-report measure administered weekly.^36^ Secondary outcomes included headache diary, depression (9-item Patient Health Questionnaire [PHQ-9]), anxiety (7-item Generalized Anxiety Disorder [GAD-7]), and sleep (Insomnia Severity Index [ISI]).^24^ Participants completed a standardized clinical interview based on the ICHD criteria for headache^37,38^ to assess qualitative headache symptoms and headache diagnosis.

Veterans with pain and/or PTSD are at high risk for treatment dropout.^39,40,41^ This study accounted for 30% dropout, and dropout participants were contacted at the end of the study to assess reason for dropout, global change in symptoms, treatment satisfaction, and the 2 primary outcomes as described earlier.^24^

### Statistical Analysis

Sample size planning assumed a large correlation between baseline scores and final end points (Pearson *r* *=* 0.50) with 2-tailed specified joint superiority testing of 2 primary outcomes at α = .025. Group sample sizes of 64 participants (total number = 192) would provide power of 0.80 to detect an effect size (*d*) of 0.52 between a treatment group and TPU for both primary outcomes. This effect size translates to a clinically significant change of 2.8 points on the HIT-6^42^ and 8.2 points on the PCL-5. The primary analysis set was intention to treat (ITT), including all individuals randomly assigned to treatment. A multiple imputation strategy was applied to account for missing data in the ITT analyses. Missing outcome scores at posttreatment, 3-month, and 6-month follow-ups were multiply imputed (m = 100) using multilevel models (Supplement 2).

Primary outcomes were analyzed using 2 separate generalized linear mixed models to examine the superiority of CBTH and CPT in alleviating headache disability (HIT-6) and PTSD symptoms (PCL-5) compared with TPU. Outcome observations at posttreatment and 3-month and 6-month follow-ups were entered simultaneously into the mixed model to provide a single inference about the outcome effect after treatment (hereafter referred to as aggregate posttreatment). Fixed effects included baseline HIT-6/PCL-5 scores and treatment group (ie, CBTH, CPT, TPU). To account for repeated measurements across posttreatment assessment occasions, a random intercept was used for participants specifying a normal distribution with identity link for both outcomes. Secondary analyses examined varying treatment response using a similar mixed model including statistical interaction (ie, treatment group × posttreatment time) and contrasted CBTH vs CPT for both primary outcomes (eTables 2 and 3 in Supplement 3).

Post hoc contrasts compared both behavioral treatments with TPU at each posttreatment measurement occasion using *P* < .025 as the threshold for 2-tailed statistical significance to account for the 2 primary end points (HIT-6, PCL-5). No multiplicity adjustments were made for secondary outcomes (eg, headache frequency/intensity, depression), which used *P* < .05 as the statistical significance threshold using the same generalized linear mixed-model approach for primary analysis described previously. Sensitivity analyses addressed missing data using maximum likelihood estimation, modified ITT, and per-protocol analysis sets. Statistical analyses were conducted using R software, version 4.0.2 (R Foundation) and R Studio (Supplement 2). Data were analyzed from January 20, 2020, to February 2, 2022.

## Results

A total of 193 post-9/11 combat veterans (mean [SD] age, 39.7 [8.4] years; 167 male veterans [87%]; 26 female veterans [13%]) were included in the study. Participants with the following race and ethnicity were included: 5 American Indian (2.6%), 7 Asian (3.6%), 34 Black/African American (17.6%), 81 Hispanic (42.0%), 110 non-Hispanic (57.0%), 6 Native Hawaiian (3.1%), 110 White (57.0%), 29 other race and ethnicity (15.1%) and 2 participants (1.0%) refused to answer. Race and ethnicity included in the other category included Asian and White, Black and Caribbean Indian, European Mix, Mexican American, Mixed, Native Texan/American, Puerto Rican, and White/Pacific Islander. The total participants were randomly assigned to either the CBTH group (65 [33.7%]), CPT group (64 [33.2%]), or TPU group (64 [33.2%]) (Figure 1). Treatment initiation rates were 85% (55 of 65 participants) CBTH, 75% (48 of 64 participants) CPT, and 89% (57 of 64 participants) TPU. Treatment engagement was better for CBTH and TPU compared with CPT with 60% (39 of 65 participants) completing 6 or more CBTH sessions, 42% (27 of 64 participants) completing 9 or more CPT sessions, and 83% (53 of 64 participants) completing TPU. Of the 193 randomly assigned individuals, 119 (61.7%) provided at least 1 posttreatment outcome assessment, and 80 (41.5%) provided complete outcome data for all follow-up assessments. ITT analysis included 193 participants, and the per-protocol analysis included 105 participants.

Most of the participants served in the Army (153 [79%]) as enlisted personnel (181 [94%]), and 94% reported service-connected disability (181 of 193) (Table 1). Participants frequently reported medical (144 [75%]) and mental health (152 [79%]) comorbidities and most reported taking medication for headache (150 [78%]) at enrollment (Table 2). Veterans entered the study approximately 2 years after initial headache onset, most with intermittent headaches (150 [78%]) occurring a mean (SD) of 3.8 (3.1) times per week, with a mean (SD) duration of 4.2 (3.9) hours and a mean (SD) intensity of 6.9 (2.0) out of 10. Overall, 120 veterans (62%) reported migraine, 23 (12%) tension-type headache, and 32 (17%) cluster headache symptoms. Twenty-seven participants (14%) reported headache duration longer than 24 hours, and 16 (8%) reported unremitting headaches.

**Table 1.** Sample Demographic Characteristics

Demographic	No. (%)			SMD
	CBTH (n = 65)	CPT (n = 64)	TPU (n = 64)	
Male gender	56 (86.2)	58 (90.6)	53 (84.1)	0.13
Female gender	9 (13.8)	6 (9.4)	10 (15.6)^a^	
BMI, mean (SD)^b^	30.72 (5.28)	31.27 (4.77)	31.24 (5.48)	0.07
Ethnicity				
Hispanic	27 (41.5)^c^	28 (43.8)	26 (40.6)^c^	0.03
Non-Hispanic	37 (57.8)	36 (56.2)	37 (58.7)	
Race				
American Indian	0 (0.0)	1 (1.6)	4 (6.3)	0.44
Asian	2 (3.1)	1 (1.6)	4 (6.3)	
Black/African American	7 (10.8)	14 (22.2)	13 (20.6)	
Native Hawaiian	2 (3.1)	2 (3.2)	2 (3.2)	
White	43 (66.2)	34 (54.0)	33 (52.4)	
Other^d^	11 (16.9)	11 (7.5)	7 (11.1)	
Marital status				
Never married	3 (4.6)	6 (9.4)	4 (6.3)	0.33
Not cohabiting	3 (4.6)	0 (0.0)	4 (6.3)	
Cohabiting	8 (12.3)	4 (6.2)	5 (7.9)	
Married	41 (63.1)	43 (67.2)	41 (65.1)	
Separated, divorced	10 (15.4)	11 (17.2)	9 (14.3)	
No. of children, mean (SD)	2.32 (2.02)	2.31 (1.82)	2.51 (1.64)	.07
Education				
Some high school	1 (1.5)	0 (0.0)	0 (0.0)	0.40
GED	0 (0.0)	0 (0.0)	2 (3.2)	
High school diploma	4 (6.2)	8 (12.5)	6 (9.5)	
Some college	25 (38.5)	25 (39.1)	23 (36.5)	
Associate degree	18 (27.7)	11 (17.2)	12 (19.0)	
4-y Degree	12 (18.5)	15 (23.4)	13 (20.6)	
Master’s degree	5 (7.7)	4 (6.2)	7 (11.1)	
Doctoral degree	0 (0.0)	1 (1.6)	0 (0.0)	
Military status				
Active duty	0 (0.0)	1 (1.6)	0 (0.0)	0.40
Reserve	2 (3.1)	0 (0.0)	0 (0.0)	
National Guard	2 (3.1)	0 (0.0)	3 (4.7)	
Veteran				
Retired	29 (44.6)	35 (54.7)	25 (39.1)	
Separated	32 (49.2)	28 (43.8)	36 (56.2)	
Military branch				
US Army	47 (72.3)	54 (84.4)	52 (81.2)	0.32
US Marine Corps	9 (13.8)	7 (10.9)	5 (7.8)	
US Air Force	6 (9.2)	2 (3.1)	5 (7.8)	
US Navy	3 (4.6)	1 (1.6)	1 (1.6)	
US Coast Guard	0 (0.0)	0 (0.0)	1 (1.6)	
Military rank				
Officer	3 (4.6)	5 (7.8)	4 (6.2)	0.09
Military service, mean (SD)				
Years	13.82 (8.15)	12.84 (7.43)	13.95 (8.84)	0.09
Months	3.68 (3.73)	3.72 (3.64)	3.05 (3.46)	
Employment				
Full-time	22 (34.4)	12 (19.0)	22 (34.4)	0.54
Part-time	7 (10.9)	7 (11.1)	3 (4.7)	
No regular employment	3 (4.7)	2 (3.2)	12 (18.8)	
Unemployed	32 (50.0)	42 (66.7)	27 (42.2)	
Income, $				
<10 000	2 (3.1)	2 (3.3)	2 (3.1)	0.30
10 000-20 000	6 (9.2)	6 (9.8)	3 (4.7)	
20 000-35 000	10 (15.4)	10 (16.4)	13 (20.3)	
35 000-50 000	20 (30.8)	14 (23.0)	17 (26.6)	
50 000-100 000	20 (30.8)	25 (41.0)	20 (31.2)	
>100 000	7 (10.8)	4 (6.6)	9 (14.1)	
VA disability rating, mean (SD)	74.60 (30.47)	85.87 (21.45)	84.69 (25.26)	.28

Abbreviations: BMI, body mass index; CBTH, cognitive behavioral therapy for headache; CPT, cognitive processing therapy; GED, general education diploma; TPU, treatment per usual; VA, US Department of Veterans Affairs.

^a^ One (1.6%) case had missing data for gender in the TPU group not reported in the Table.

^b^ Calculated as weight in kilograms divided by height in meters squared.

^c^ There was 1 missing value for CBTH and TPU under Hispanic ethnicity that was not reported in the Table.

^d^ Other indicates Asian and White, Black and Caribbean Indian, European Mix, Mexican American, Mixed, Native Texan/American, Puerto Rican, and White/Pacific Islander.

**Table 2.** Baseline Comorbidities and Headache Treatments

Comorbid condition	No. (%)^a^			SMD
	CBTH (n = 65)	CPT (n = 64)	TPU (n = 64)	
Any medical condition	51 (78.5)	46 (73.0)	47 (73.4)	0.09
Hypertension	44 (71.0)	44 (75.9)	43 (74.1)	0.07
High cholesterol	14 (22.6)	16 (27.6)	17 (29.3)	0.10
Heart disease	1 (1.6)	2 (3.4)	0 (0.0)	0.19
Asthma	6 (9.7)	5 (8.6)	7 (12.1)	0.08
Sleep apnea	24 (38.7)	26 (44.8)	23 (39.7)	0.08
Type 2 diabetes	4 (6.5)	8 (13.8)	9 (15.5)	0.20
Thyroid	1 (1.6)	1 (1.7)	0 (0.0)	0.13
Amputation	0 (0.0)	1 (1.7)	0 (0.0)	0.13
Any mental health condition	50 (76.9)	49 (76.6)	53 (82.8)	0.10
Depression	31 (50.8)	37 (61.7)	31 (48.4)	0.18
Generalized anxiety	28 (54.9)	31 (51.7)	28 (43.8)	0.11
Panic	1 (1.6)	2 (3.3)	2 (3.1)	0.07
PTSD	49 (80.3)	55 (91.7)	55 (85.9)	0.22
Bipolar disorder	1 (1.6)	3 (5.0)	1 (1.6)	0.13
Alcohol use disorder	0 (0.0)	2 (3.3)	0 (0.0)	0.19
Psychosis	0 (0.0)	2 (3.3)	0 (0.0)	0.18
Active medications	48 (73.8)	50 (78.1)	51 (81.0)	0.11
Pain, nonopioid	13 (37.1)	14 (35.0)	10 (34.5)	0.04
SSRI	10 (15.4)	22 (34.4)	20 (31.2)	0.30
SNRI	4 (6.2)	8 (12.5)	12 (18.8)	0.26
Sleep	23 (35.4)	19 (29.7)	27 (42.2)	0.18
Antipsychotic	1 (1.5)	5 (7.8)	5 (7.8)	0.05
Benzodiazepine	13 (20.0)	22 (34.4)	20 (31.2)	0.22
Opioid	7 (10.8)	12 (18.8)	9 (14.1)	0.15
Gabapentin	4 (6.2)	13 (20.3)	10 (15.6)	0.29
Blood pressure	16 (24.6)	17 (26.6)	18 (28.1)	0.05
Cholesterol	4 (6.2)	11 (17.2)	8 (12.5)	0.23
Type 2 diabetes	1 (1.5)	5 (7.8)	4 (6.2)	0.20
Topiramate	2 (3.1)	10 (15.6)	7 (10.9)	0.30

Abbreviations: CBTH, cognitive behavioral therapy for headache; CPT, cognitive processing therapy; PTSD, posttraumatic stress disorder; SMD, standardized mean difference; SNRI, serotonin-norepinephrine reuptake inhibitor; SSRI, selective serotonin reuptake inhibitor; TPU, treatment per usual.

^a^ Percentage is based on nonmissing data.

### Primary Outcomes

#### Headache Disability

**Figure 2.**
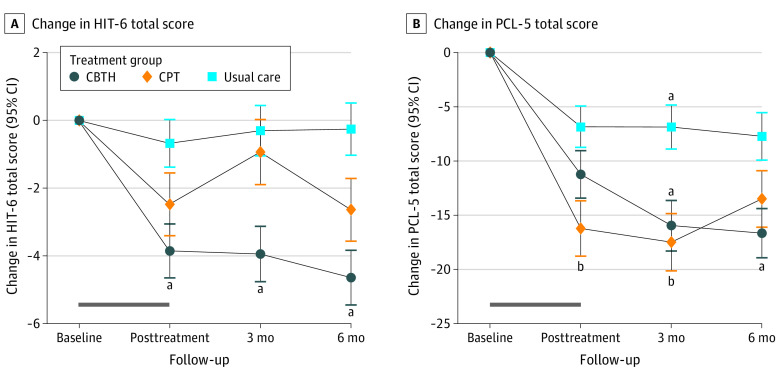
Change in 6-Item Headache Impact Test (HIT-6) and Posttraumatic Stress Disorder Checklist for *DSM-5 *(PCL-5) for the Cognitive Behavioral Therapy for Headache (CBTH), Cognitive Processing Therapy (CPT), and Treatment per Usual (TPU) Study Groups The contrasts reflect the mean (SE) change in HIT-6 (A) and PCL-5 (B) scores for that group from their baseline values. Plotted values are the observed data based on intention to treat. ^a^Statistically significant difference between CBTH and TPU at that assessment time point. ^b^Statistically significant difference between CPT and TPU at that assessment time point.

Participants reported high levels of baseline headache-related disability (mean [SD]: CBTH, 66.1 [5.4]; CPT, 66.1 [5.1]; TPU, 65.2 [6.4]). Veterans reported severe baseline headache-related disability (mean [SD] HIT-6 score, 65.8 [5.6] points) and severe PTSD symptoms (mean [SD] PCL-5 score, 48.4 [14.2] points). Based on ITT analysis, participants randomly assigned to CBTH reported significantly lower aggregate posttreatment mean HIT-6 scores compared with TPU (−3.4; 95% CI, −5.4 to −1.4; *P* < .01), but the posttreatment effect for CPT compared with TPU was modest (−1.4; 95% CI, −3.7 to 0.8; *P* = .21) (Table 3 and Figure 2A). Aggregate posttreatment HIT-6 score estimates were stable throughout posttreatment measurement occasions (group × time interaction, *P* = .47) with the posttreatment contrast between CBTH and TPU decreasing −0.5 units (95% CI, −2.9 to 1.9) at 3 months and −1.7 units (95% CI, −4.1 to 0.7) at 6 months whereas the posttreatment contrast between CPT and TPU increased 1.2 units (95% CI, −1.4 to 3.8) at 3 months and decreased −0.7 units (95% CI −3.4 to 2.0) at 6 months. Aggregate estimates were somewhat attenuated after multiple imputation to replace missing data, CBTH vs TPU, −2.4 (95% CI, −4.1 to −0.7; *P* = .01) and CPT vs TPU, −1.0 (95% CI, −2.8 to 0.8; *P* = .30). When the active treatments were compared with one another, CBTH demonstrated lower aggregate posttreatment HIT-6 scores compared with CPT (reference group), but the difference did not reach statistical significance (−2.0; 95% CI, −4.3 to 0.3; *P* = .09).

**Table 3.** Aggregate Intent-to-Treat Primary and Secondary Outcomes for CBTH and CPT Compared With TPU

Outcome	Time	TPU (n = 64)	CBTH (n = 65)		CPT (n = 64)	
			Treatment contrasts (95% CI)	*P* value	Treatment contrasts (95% CI)	*P* value
**Primary outcomes^a^**						
HIT-6	All posttreatment^b^	NA	−3.4 (−5.4 to −1.4)	<.001	−1.4 (−3.7 to 0.8)	.21
PCL-5			−6.5 (−12.7 to −0.3)	.04	−8.9 (−15.9 to −1.9)	.01
**Secondary outcomes^c^**						
Headache intensity	All posttreatment^b^	NA	−0.6 (−1.3 to 0.1)	.11	−0.3 (−1.1 to 0.5)	.47
Headache frequency			−2.9 (−6.0 to 0.1)	.07	−2.1 (−5.6 to 1.4)	.26
Depression (PHQ-9)			−1.7 (−3.4 to 0.0)	.05	−1.2 (−3.1 to 0.7)	.23
Anxiety (GAD-7)			−1.1 (−2.9 to 0.6)	.20	−1.8 (−3.8 to 0.1)	.07
Insomnia (ISI)			−0.8 (−3.0 to 1.4)	.47	−2.0 (−4.5 to 0.5)	.12

Abbreviations: CBTH, cognitive behavioral therapy for headache; CPT, cognitive processing therapy; GAD-7, 7-item Generalized Anxiety Disorder screening tool; HIT-6, 6-Item Headache Impact Test; ISI, Insomnia Severity Index; NA, not applicable; PCL-5, Posttraumatic Stress Disorder Checklist for *DSM-5*; PHQ-9, 9-item Patient Health Questionnaire depression screening tool; TPU, treatment per usual.

^a^ Primary outcome statistical significance threshold adjusted to *P* = .025.

^b^ All posttreatment = aggregate effect summary of posttreatment and 3-month follow-up and 6-month follow-up combined into a single effect.

^c^ Secondary outcome statistical significance threshold at *P* = .05. Headache intensity/frequency were derived from headache diary. Treatment contrasts (95% CI) are reported that compare each active treatment group with treatment per usual.

Examination of disaggregated posttreatment outcomes for the 2 treatment groups showed that participants in the CBTH group demonstrated a mean (SE) HIT-6 score decrease of −3.9 (0.8) units from baseline to posttreatment, which remained stable at 3-month follow-up (−3.9 [0.8] units) and further decreased to −4.6 (0.8) units at 6-month follow-up. Participants randomly assigned to CPT demonstrated a mean (SE) HIT-6 score decrease of −2.5 (0.9) units from baseline to posttreatment, which rose to −0.9 (1.0) units at 3-month follow-up but decreased again at 6-month follow-up to −2.6 (0.9) units. There was minimal change (<1 unit) in mean HIT-6 score from baseline for participants who received TPU (eTable 4 in Supplement 3).

#### Posttraumatic Stress Disorder

Participants in all groups reported high mean (SD) PCL-5 scores at baseline (CBTH, 47.7 [14.7]; CPT, 48.6 [14.6]; TPU, 49.0 [13.3]); 85% met diagnostic criteria for PTSD based on blinded Clinician-Administered PTSD Scale for *DSM-5* interview. Compared with participants in the TPU group, participants in the CBTH group reported lower mean aggregate posttreatment PCL-5 scores that did not reach statistical significance (−6.5; 95% CI, −12.7 to −0.3; *P* = .04), but the difference between participants randomly assigned to CPT compared with those assigned to TPU was statistically significant (−8.9; 95% CI, −15.9 to −1.9; *P* = .01). These estimates were somewhat changed after multiple imputation, CBTH vs TPU (−5.6; 95% CI, −10.6 to −0.6; *P* = .04) and CPT vs TPU (−6.0; 95% CI, −10.0 to −0.2; *P* = .03). Treatment effect exhibited some variability throughout posttreatment measurement occasions, though not statistically significant (treatment group × time interaction, *F*_4,168.9 _= 2.3; *P* = .06) (Figure 2B and Table 3). The difference in PCL-5 score between CBTH and TPU decreased −4.1 points (95% CI, −9.7 to 1.5) from posttreatment to 3 months with a further −4.9 point decrease (95% CI, −10.7 to 1.0) at 6 months. The posttreatment difference between CPT and TPU decreased −0.7 points (95% CI, −6.8 to 5.3) from posttreatment to 3 months with an increase of 4.5 points (95% CI, −1.7 to 10.8) at 6 months. Compared with one another, there was no significant difference in aggregate posttreatment PCL-5 score between CBTH and CPT (2.4; 95% CI, −4.9 to 9.7; *P* = .52).

Examination of disaggregated PCL-5 outcomes revealed that participants in the CBTH group demonstrated a mean (SE) decrease in PCL-5 score of −11.2 (2.2) points from baseline to posttreatment, which further decreased to −16.0 (2.3) points at 3-month follow-up and −16.7 (2.3) points at 6-month follow-up. Participants randomly assigned to CPT demonstrated a mean (SE) PCL-5 score decrease of −16.2 (2.6) points from baseline to posttreatment, with a decrease of −17.5 (2.6) points at 3-month follow-up and −13.5 (2.6) points at 6-month follow-up. Participants in the TPU group reported a mean (SE) PCL-5 score decrease of −6.8 (1.9) points from baseline to posttreatment which remained stable at 3-month (−6.9 [2.0] points) and 6-month (−7.7 [2.2] points) follow-ups (eTable 4 in Supplement 3).

### Secondary Outcomes

No statistically significant differences in secondary outcomes were observed (Table 3). Compared with participants in the TPU group, those in both the CBTH and CPT groups reported an aggregate posttreatment difference in headache intensity of less than 1 point and a difference in headache frequency of −2.9 (95% CI, −6.0 to 0.1; *P* = .07) headache days per month for CBTH and −2.1 (95% CI, −5.6 to 1.4; *P* = .26) for CPT. Measures of depression (PHQ-9), anxiety (GAD-7), and insomnia (ISI) showed no significant posttreatment difference compared to TPU for either CBTH or CPT (eTable 5, eFigures 1-5 in Supplement 3).

### Treatment Fidelity and Adverse Events

Independent research staff randomly reviewed 10% of all treatment session audio recordings and found that CBTH and CPT therapists achieved over 89% treatment adherence. Adverse event data (n = 160) showed that most adverse events (eg, changes in headache/musculoskeletal pain, increased stress, depression) were not study related. Two participants in the CPT group reported temporary exacerbation of PTSD symptoms attributed to trial participation (eTables 6-9 in Supplement 3).

## Discussion

The present randomized clinical trial enrolled a PTH sample of participants with comorbid PTSD symptoms and found a significant improvement in headache-related disability among those treated with CBTH. PTH attributable to mTBI is complex, prevalent among military veterans, and highly disabling.^43^ A previous treatment study concluded that nonpharmacological interventions (ie, CBT) may not work for individuals with PTH.^19^ However, the present trial offers the first evidence showing that a nonpharmacological intervention (CBTH) can significantly improve headache-related disability in PTH attributable to mTBI for up to 6 months compared with usual care. PTSD has been theorized as a potential mechanism of PTH in military veterans,^44,45^ but CPT failed to improve headache disability in this trial despite significant reductions in PTSD symptom severity. This finding suggests that PTSD and PTH may be independent in this population. Notably, CBTH completers demonstrated significant improvement over usual care for headache disability and clinically significant PTSD symptom improvement with treatment effects lasting up to 6 months posttreatment.

Secondary analyses did not find a significant improvement in headache frequency or intensity despite improvements in HIT-6 scores, which was surprising because headache frequency and intensity are strong predictors of disability in studies of migraine^46^ and tension-type headache.^47^ Behavioral interventions like CBTH are better suited to address disability than headache frequency or intensity, especially when headaches are chronic and pain cognitions/behaviors (the primary targets of CBT treatment) may be more related to function than pain experience.^48^ Indeed, studies show that interventions such as CBTH have questionable effect on headache frequency and headache days,^22,49^ though little research has examined these treatments for PTH.^18^

More than 40% of individuals with PTH experience comorbid PTSD,^12^ which is linked to severe headache intensity and disability.^50,51^ Although not statistically significant compared with TPU, participants in the CBTH group demonstrated remarkable PTSD symptom improvement over time with fewer treatment sessions and less clinician training than CPT, and CBTH outcomes on the PCL-5 exceeded the threshold for reliable change for male military veterans with PTSD.^52^ Recent studies show that brief psychotherapies like CBTH can produce PTSD outcomes noninferior to criterion-standard treatments like CPT.^53^ In the present study, there was significantly higher dropout in the CPT group compared with the CBTH and TPU groups; therefore, better PTSD outcomes in CBTH may be attributable to higher rates of treatment completion. Military trauma survivors drop out from trauma-focused treatments at high rates,^54^ and CBTH participants may have completed more treatment because these treatments do not address trauma symptoms like CPT does. Cognitive-behavioral headache interventions improve coping self-efficacy,^38^ which may also indirectly improve PTSD.^55^

Clinical benefit from nonpharmacological interventions is dose dependent,^56^ and treatment dropout is common in veterans treated for PTSD and pain. Treatment initiation was 75% or better in both behavioral treatment groups, with more than 50% completion rates (better than expected based on strict timelines for completing treatment). PTSD clinical trials report high dropout rates,^57^ and veterans drop out of behavioral treatments for any chronic condition at high rates (>30%).^58,59^ Mild TBI increases dropout risk,^58,60^ possibly owing to exacerbation of PTSD-related physical symptoms,^61^ such as headache.^62,63^ Study staff interviewed approximately one-third of study dropouts, and found dropout was primarily attributable to logistical factors. Most contacted CBTH dropouts reported time/travel as their reason for dropout and 2 found CBTH too difficult. More than half of CPT dropouts reported time/travel as an obstacle, and some found CPT too difficult. In both behavioral groups, dropouts receiving some treatment reported moderate satisfaction and low burden.

### Limitations

Trial limitations include the chosen primary outcome, blinding a behavioral trial, definition of PTH as headache onset within 3 months of head injury (instead of 7 days per ICHD-3 5.2.2), and missing data/dropout. The HIT-6 was chosen at trial registration because of the established literature supporting its use in studies of behavioral interventions for headache^35^ and the relevance of disability in headache attributable to TBI with comorbid PTSD.^12^ The present study was powered to detect a HIT-6 total score change of 2.8 points based on a minimally important change threshold established in a primary care sample of migraine patients with an average of 6 headache days per month.^42^ The present sample was more severe than the primary care migraine sample; therefore, the chosen threshold of clinically significant change may not apply.

To our knowledge, there is no established minimally important change threshold for the HIT-6 in headache attributable to mild TBI. Studies of chronic migraine samples found that HIT-6 total score decrease of 2.3 to 3.7 units represented at least somewhat better headache. However, a change threshold of up to 6 units may be needed for meaningful clinical change in more severe populations based on comparisons between pharmacological interventions to placebo.^64,65,66^ The present study compared a behavioral treatment with comprehensive usual care; therefore, the threshold of change established in placebo-controlled studies may not apply. Headache intensity and frequency did not significantly improve despite changes in HIT-6 total score. This discrepancy may be attributable to better sensitivity to change in self-reported disability for nonpharmacological pain treatments compared with pain diaries,^48^ although there is scant research on the suitability of any outcome for PTH attributable to mTBI.

Blinding a behavioral trial is complex and may bias outcomes. Assessments were administered by trained assessors blind to the randomized condition of the participant and treatment professionals were blind to assessments and study hypotheses. We expanded the headache onset criterion to 3 months after head injury (per ICHD-3 A5.2.2.1),^67^ and our research group recently found no phenotypic difference between veterans with headache onset within 7 days (ICHD-3 5.2.2) vs 8 to 90 days (A5.2.2.1) after head injury.^67^ Thus, the present sample reasonably reflects veterans with persistent PTH, including those misdiagnosed owing to headache onset greater than 7 days.

Dropout and missing data from this trial were high, though consistent with dropout risk in this population. We developed a priori strategies to manage missing data, including planned sensitivity analyses and contact with dropout participants to assess reasons for dropout. Sensitivity analyses reached similar conclusions to our primary analyses, although withdrawals by patient request were not evenly distributed across the 3 groups suggesting possible bias in dropout. On exit interview, most patients who requested withdrawal reported lack of time or transportation as their primary barrier, which reinforces time burden as an obstacle to treatment/study completion.

## Conclusions

Results of this randomized clinical trial found that US military combat veterans with PTH attributable to mTBI and comorbid PTSD symptoms showed significant improvement in headache-related disability and PTSD symptom severity in response to nonpharmacological interventions for headache and PTSD. The CBTH intervention successfully addressed headache with unexpected improvement in PTSD symptoms, whereas CPT only successfully addressed PTSD symptom severity. The present study provided evidence supporting treatment of PTH disability using a manualized headache intervention, with outcomes superior to multimodal usual care. Notably, the headache intervention also showed promise in addressing PTSD symptoms, but further research is needed to explore how this treatment influences PTH and PTSD, explore dissemination, and examine if integrated CBTH and CPT can improve outcomes.

## Notes

Supplement 1. Trial Protocol

Supplement 2. Statistical Analysis Plan

Supplement 3. **eTable 1.** Treatment Elements **eTable 2.** ANOVA for the Primary Analyses **eTable 3.** Treatment × Postintervention Time (1, 3, 6 months) **eTable 4.** Primary Outcomes and Sensitivity Analyses **eTable 5.** Descriptive Statistics for Primary and Secondary Outcomes **eFigure 1.** GAD-7 **eFigure 2.** Insomnia Severity Index (ISI) **eFigure 3.** PHQ-9 **eFigure 4.** Headache Frequency (28-day month) **eFigure 5.** Headache Intensity (0-10) **eTable 6.** Secondary Outcome Plots Model Contrasts **eTable 7.** Multiple Imputation **eTable 8.** Results of the Pooled Estimates **eTable 9.** Effect Plot

Supplement 4. Data Sharing Statement
