# Supplementary material for: Cognitive Behavioral Therapy for Veterans With Comorbid Posttraumatic Headache and Posttraumatic Stress Disorder Symptoms: A Randomized Clinical Trial
Source: JAMA Neurol. 2022 Jun 27;79(8):746–57. doi: 10.1001/jamaneurol.2022.1567 (PMC9237802; doi:10.1001/jamaneurol.2022.1567)
Supplement: Supplement 1. — Trial Protocol [file jamaneurol-e221567-s001.pdf]

1. PROTOCOL TITLE: Randomized Clinical Trial of Cognitive-Behavior Therapy for Posttraumatic Headache

2. ABSTRACT: The purpose of this randomized clinical trial of 192 military Veterans with posttraumatic headache (PTHA) and co-morbid symptoms of posttraumatic stress is to compare an 8-session manualized clinic-based cognitive behavioral therapy (CCBT) headache intervention delivered over 6 weeks, to a 12-session manualized Cognitive Processing Therapy – Cognitive only (CPT-C) for PTS delivered over 6 weeks, to Treatment As Usual (TAU) in the relief of both PTHA and PTS symptoms following therapy and at 3- and 6-months.

3. OBJECTIVES/SPECIFIC AIMS/RESEARCH QUESTIONS: The overall purpose of the study is to compare two talk therapies (CCBT and CPT-C) for the treatment of posttraumatic headache (PTHA) and co-morbid posttraumatic stress (PTS). The researchers hope to learn if a non-medication, cognitive behavioral treatment can result in noticeable reductions in PTHA intensity/severity and frequency as well as PTS symptom severity.

4. BACKGROUND AND SIGNIFICANCE

Posttraumatic headache (PTHA) is a singularly unique and vexing by-product of traumatic brain injury (TBI) offering numerous challenges both to the individuals experiencing them as well as the medical providers tasked with treating this growing problem (Monteith et al., 2009). Although PTHA is not a new phenomenon (there are reports of posttraumatic headache dating back to the 1700s), very little is known about headaches with onset or exacerbation after TBI.

One of the greatest problems facing posttraumatic headache research is inconsistency in how PTHAs are defined. The most widely recognized definition is listed in the International Classification of Headache Disorders (ICHD-II), assembled by the International Headache Society. The ICHD-III (beta) criteria classify PTHA as a secondary headache attributed to head and/or neck trauma. PTHA symptoms can present similarly to primary headache conditions like migraine or tension- type headache, but the onset or exacerbation of the headache symptoms must be secondary to trauma exposure. Acute versus chronic PTHA is identified based on the duration and persistence of the posttraumatic headache. Posttraumatic headaches that resolve within 3 months, or are present in individuals with trauma exposure less than 3 months ago, are classified as acute. If posttraumatic headache persists beyond 3 months, then it is considered chronic. There is also widespread agreement that PTHA goes beyond de novo headaches, including marked worsening of premorbid headache symptoms attributable to a traumatic event.

Despite the significant incidence of PTHA among individuals who suffer TBI, very little is known about its treatment. To date, there are no controlled studies of PTHA treatment, and the nesting of PTHA symptoms within postconcussion syndrome makes this population unresponsive to typical primary headache medications. Many PTHA sufferers attempt self-treatment through the use of acetaminophen, ibuprofen, and opiate/triptan medication (usually prescribed for other reasons). Unfortunately, these over-the-counter or self-prescribed medications do not effectively address PTHA symptoms and may result in headache worsening through medication overuse. Over one-third of PTHA patients are referred to Neurology, where they are typically prescribed anticonvulsants, tricyclics, or gabapentin. Patients with PTHA referred to Neurology usually present with more severe and persistent PTHA symptoms, and few benefit from Neurology intervention. Less than one-third of those referred to Neurology actually present for their first appointment, and 40% of those who attend a first appointment fail to return for subsequent appointments. As a result, there are few studies of medication treatment in PTHA, and many patients refuse medication interventions due to concerns about headache worsening with medication overuse, inability to complete follow-up due to mTBI and PTHA disability, dislike of medications and side effects, and general mistrust of the medical system. Based on significant concerns about medical treatments and poor response to medical interventions, it is strongly recommended that PTHA be addressed through a comprehensive multidisciplinary treatment program offering evidence-based, non-medical interventions for headache management.

Psychiatric comorbidities are common in headache (Breslau et al., 2003), and comorbid psychopathology has been linked to alterations in headache evolution and treatment response (Antonaci et al., 2011). Ruff et al. (2012) estimate that more than 80% of Veterans diagnosed with mTBI and PTHA also have PTSD. Although comorbid headache and PTSD may alter the effectiveness of interventions, there is some evidence to suggest that addressing PTSD directly can ameliorate health symptoms like headache (Shipherd et al., 2013). There are no studies, however, examining the influence of PTSD comorbidity in posttraumatic headache in a military population, so the gaps in PTHA intervention extend beyond targeted non- medication interventions to PTSD treatments that address PTHA through alterations in PTSD symptom severity. In fact, an ongoing PTSD trial overseen by Dr. Patricia Resick, has revealed subjective reports of headache improvement among military service members who are successfully treated for PTSD. We have chosen to add a treatment arm to this study design in which participants with PTHA and comorbid PTSD or PTS symptoms will be treated with a gold-standard, manualized PTSD intervention (Cognitive Processing Therapy; CPT-C). Both CPT-C and the manualized headache intervention rely upon cognitive behavioral mechanisms that overlap and may produce relief for both disorders.

5. RESEARCH DESIGN

Due to the lack of studies exploring behavioral interventions for PTHA, this research is designed as a randomized clinical trial comparing an 8-session manualized CBT intervention for headaches (CCBT) to the 12 session Cognitive Processing Therapy – Cognitive Only (CPT-C) to Treatment as Usual (TAU) for patients with co-morbid symptoms of PTHA and posttraumatic stress following military deployment and combat trauma. The resulting data will significantly add to the extant research by gathering preliminary evidence supporting the efficacy of a manualized migraine headache intervention in 192 post 9/11 military Veteran and active duty Service Members with PTHA. Assessments will occur prior to treatment and 1-, 3-, and 6- months (following treatment completion). Blood will be collected prior to treatment, during two of the sessions of the 6- week treatment phase for all participants and at 1- and 6-months following treatment in collaboration with the CAP Genomics and Basic Science Core (Douglas E. Williamson, Ph.D. – PI) to examine gene expression profiles links with PTHA that may be predictive of treatment outcomes. Veterans randomized to the TAU group will have an option to be treated clinically with either CCBT or CPT-C after their 6-month follow-up assessment is complete.

6. RESEARCH PLAN

6.1 Selection of Subjects

6.1.1. Subject Population. Potential participants will be recruited from the STVHCS Polytrauma Rehabilitation Center (PRC), military medical treatment facilities, and from the local community. Recruitment will target veterans and active duty personnel having returned from a post-9/11 deployment with chronic PTHA and symptoms of posttraumatic stress.

6.1.2. Source of Research Material. Please find a description of these measures at the end of this protocol. Assessments will be administered in person whenever possible. However, in order to accommodate participant schedules and/or instances in which a participant may have left the local area at the time of a follow up assessment, we may collect full or partial assessments in person or via phone or electronic data capture using a secure link to the encrypted STRONG STAR database. Assessments completed via phone or remote electronic data capture will not include biospecimen collection. Reasonable efforts will be made to collect all data as described in this protocol, but we expect some participants may not be able to complete part or all of any given follow up assessment.

Assessments	PC/RA or IE	BL	TX	Post	3MFU	6MFU	DC
**Demographics and Military Questionnaire**	PC/RA	x					
**History of Head Injuries and History of Head Injuries Addendum**	PC/RA	x		x	x	x	
**Deployment Risk and Resiliency Inventory_2_Deployment Environment (DRRI_2_D)**	PC/RA	x					
**Deployment Risk and Resiliency Inventory_2_Postbattle Experiences (DRRI_2_P)**	PC/RA	x					
**Deployment Risk and Resiliency Inventory_2_Combat Experiences (DRRI_2_C)**	PC/RA	x					
**Life Events Checklist-5 (LEC-5)**	PC/RA	x		x	x	x	
**Patient Health Questionnaire-9 (PHQ-9)**	PC/RA	x	x	x	x	x	
**Depressive Symptom Index –Suicide Subscale (DSI-SS)**	PC/RA	x		x	x	x	
**Generalized Anxiety Disorder-7 (GAD-7)**	PC/RA	x		x	x	x	
**Alcohol Use Disorders Identification Test –Self Report (AUDIT)**	PC/RA	x					
**Quick Drinking Screen (QDS)**	PC/RA	x		x	x	x	
**Fagerstrom Test for Nicotine Dependence (FTND)**	Self-report	x		x	x	x	
**Fagerstrom Test for Nicotine Dependence-Smokeless Tobacco (FTND-ST)**	Self-report	x		x	x	x	
**Headache Management Self-efficacy Scale (HMSE)**	PC/RA	x		x	x	x	
**Headache-Specific Locus of Control Scale (HSLC)**	PC/RA	x		x	x	x	
**Headache Impact Test (HIT-6)**	PC/RA	x		x	x	x	x
**Neurobehavioral Symptom Inventory (NSI)**	PC/RA	x		x	x	x	
**Credibility and Expectancy Questionnaire (CEQ)**	PC/RA		x	x			
**Pre-Treatment Health Interview (+ BL PTHA addendum)**	IE	x					
**Post-Treatment Health Interview (+ FU PTHA addendum)**	IE			x	x	x	
**Structured Diagnostic Interview for Headache –Revised (SDIH-R)**	IE	x		x	x	x	
**Ohio State University TBI Identification Method-Interview Form (OSU TBI-ID-SF)**	IE	x					
**Selection of Index Event for the CAPS-5**	IE	x					
**Clinician Administered PTSD Scale-5 (CAPS-5)**	IE	x		x	x	x	
**Self-Injurious Thoughts and Behaviors Interview –Short Form**	IE	x		x	x	x	
**Tinnitus Functional Index**	IE	x		x	x	x	
**PTSD Checklist List-5 (PCL-5)**	PC/RA	x	x	x	x	x	x
**Cognitive Emotions Regulations Questionnaire –Short (CERQ-Short)**	PC/RA	x		x	x	x	
**Posttraumatic Cognitions Inventory (PTCI)**	PC/RA	x		x	x	x	
**Response to Stressful Experiences Scale (RSES)**	PC/RA	x					
**Patient Health Questionnaire-15 (PHQ-15)**	PC/RA	x		x	x	x	
**Veterans Rand 12 Item Health Survey (VR-12)**	PC/RA	x		x	x	x	
**PROMIS Sexual Function**	PC/RA	x		x	x	x	
**Brief-Inventory of Psychosocial Functioning (B-IPF)**	PC/RA	x		x	x	x	
**Insomnia Severity Index (ISI)**	PC/RA	x		x	x	x	
**PROMIS Sleep-Related Impairment and Disturbance**	PC/RA	x		x	x	x	

**Assessments (cont.)**	**PC/RA or IE**	**BL**	**TX**	**Post**	**3MFU**	**6MFU**	**DC**
**Snoring, Tired, Observed, Blood Pressure (STOP)**	PC/RA	x					
**Community Reintegration of Injured Service Members (CRIS)**	PC/RA	x		x	x	x	
**NIH Cognition Battery (via computer)**	PC/RA	x		x	x	x	
**PTHA Study Missing Data Assessment**	PC/RA						x
**Online Forms:** **1.) Daily Headache Dairy 2.) Profile of Mood States 3.) Sleep Quality/Quantity 4.) Daily Stress Inventory**	Participant will complete online entries twice daily. Entries will occur before, during, and after treatment for a total of 14 weeks.						

* DC = participant discontinued participation (i.e., drop out of treatment or follow-up)

6.1.3. Inclusion and Exclusion Criteria.

Inclusion Criteria:

- adult (ages 18 and above)
- U.S. military Veterans and active duty personnel with military service during a post-9/11 deployment
- have sustained a traumatic head injury.
- have been diagnosed or report symptoms consistent with chronic (> 3 months) posttraumatic headache attributed to a traumatic injury. We are focusing on chronic PTHA due to the very low likelihood of headache remission after 3 months, the disability associated with chronic PTHA, and the high prevalence of chronic versus acute PTHA in this population. A positive PTHA diagnosis will be indicated for individuals with de novo headache onset after a concussion or exacerbation of pre-existing headache symptoms (increased frequency, duration, or intensity), which is consistent with the existing ICHD-IV diagnostic criteria for PTHA. PTHA inclusion will be based on a consultation with one of our PRC/Polytrauma System of Care (PSC) co-PIs/overall study PI if symptoms consistent with chronic PTHA are reported, but the diagnosis is not already documented in the participant's medical record.
- If taking headache medication, participant must agree to work with their prescriber to remain on stable doses of any prescribed headache medications for the duration of the intervention and through the follow-ups as much as possible and as medically indicated..
- Participants must also report on the Clinician Administered PTSD Scale (CAPS-5) an exposure to a traumatic event (Criterion A) and at least one intrusion symptom (Criterion B). There is some evidence suggesting 40% comorbidity between PTSD and new onset headache, so it is reasonable to assume that at least half of all PTHA participants recruited for this study will have PTHA and comorbid PTS symptoms. The inclusion of PTS symptoms in this sample is vital based on reports indicating that PTS symptoms and PTSD actually increase vulnerability to PTHA and chronic headache in military populations.

Exclusion Criteria:

- Participant has had a recent and significant change in the nature of headache symptoms over the last 6 weeks prior to screening (as determined by the investigators).
- Participants currently in CPT or prolonged exposure for PTSD.
- Participant has medication overuse headache as guided by the Structured Diagnostic Headache Interview-Revised (Brief Version; SDIH-R) and clinical judgment.
- Participant is unable to read or speak English at a 6th grade level.
- Participant has had a psychiatric hospitalization in the last 6 months.
- Participant is pregnant or plans to become pregnant during the trial (due to concerns about pregnancy-induced headache that may obscure findings)
- Participant has a present psychiatric problem that warrants immediate treatment as indicated in CPRS, flagged by an IE during evaluation, or confirmed by a clinician through screening or review of CPRS notes.
- Participant demonstrates significant cognitive impairment that could impact treatment adherence/benefit.

6.1.4. Description of the Recruitment and Prescreening Process.
Initial contact will be made through various mechanisms. Under an IRB approved HIPAA Waiver, Drs. Jaramillo, Eapen or study staff will check the VA Computerized Patient Record System (CPRS) appointment schedules and examine CPRS medical records of STVHCS patients with upcoming appointments to help identify candidates that may benefit and qualify for the study. In this case, a potentially "qualified" candidate is a PRC patient presenting

with a primary diagnosis of headache with a history of diagnosis for TBI and/or persistent post-concussive symptoms (e.g., tinnitus, dizziness) as noted in their CPRS record. Dr. Jaramillo, Dr. Eapen, or study staff will contact the veteran's provider for the pending visit to inform them that their patient may qualify for the study. Patients will be given information about the study from their medical provider, and interested patients will be consented by study staff who are housed in the PRC (the provider will notify the study staff of the potential participant). Providers will be informed about the study through In-Service presentations twice a month and will be given approved fliers describing the study and inclusion criteria. If study staff are unavailable to meet with potential participants at that time, interested patients will give permission for study staff to contact them by signing a consent to contact form. Providers in all the clinics will be briefed on study goals and the inclusion/exclusion criteria to help guide their referrals.

Study Investigators will consult with military treatment providers who may see patients that could benefit from the treatment provided by the study, and the study team may accept direct referrals from these providers. Individuals may also be referred by other STRONG STAR studies. Potential participants may also self-refer in response to recruitment information on the STRONG STAR website at www.strongstar.org. Interested persons can call or walk in to the STRONG STAR offices. STRONG STAR also recruits using various social media sites (e.g. Facebook, Twitter, LinkedIn, etc.) and web search engines (e.g. Google Ads, Bing, etc.). In addition, there may be events where information about STRONG STAR studies is provided and those interested may fill out a "consent to contact" form indicating that they would like a member of the research team to contact them at a later date to learn more about the study and schedule or complete pre-screening. Research staff will field incoming phone calls and walk-ins.

Interested individuals may directly contact the study coordinator on their own accord in response to recruitment materials distributed at the PRC/PSC clinics and locations in the local community frequented by veterans and service members, including military facilities. Appearances by the study staff in public media may also generate self-referrals. Study staff will also review CPRS medical records of community referrals (if available) before consent to aid in pre-screening and determining eligibility. Recruitment letters will be mailed to veterans or SMs registered at the STVHCS with a history of OEF/OIF/OND tour(s) through collaboration with the local VA OIF/OEF/OND program office or through research registry databases (or other open research studies) of willing Veteran or SM research participants who have consented to be contacted about further study opportunities. Recruitment materials will provide information about the study and contact information for both the study PI and study coordinator.

Individuals interested in participating will be given information about the study including inclusion and exclusion criteria and they will be screened using the telephone pre-screen questionnaire to determine the possible presence of chronic PTHA, the presence of PTS symptoms, and any indicators that would exclude them from participation. Interested and qualified participants will then get additional information about the study and will meet with the study coordinator to be formally consented into the study and undergo a baseline assessment.

6.1.5. Consent Process.
The IRB Policy on Informed Consent Process and Informed Consent Documentation will be followed. Potential participants will have the study explained to them by a trained research staff member in a safe and private location. The potential participant will be given a copy of the informed consent document (ICD) to read. After the individual has read the ICD they will be given the opportunity to take the consent home to discuss the research with family and friends. The Research Team will be available to answer any questions about the research. Once the potential participant has reached a decision, the advising staff member will go over the risks and benefits of the study and ensure the subject understands the research. The advising staff member will have the individual sign the consent form. A copy of the signed ICD will be given to the participant. The advising staff member will document the informed consent process in the medical record of veteran participants per VA guidelines. Participants who discontinue participation before completing study treatment and follow-up assessments will be contacted by phone to complete a brief assessment of their symptoms and their reason(s) for discontinuing participation. Consent for this assessment will be done verbally over the phone to prevent the need for an in-person visit for the veteran.

6.1.6. Subject Screening Procedures.
Baseline assessment will occur following consent. Following the baseline assessment, participants found to meet the inclusion criteria for the study will be randomized into the study. Participants not meeting study inclusion criteria

will be informed and referred to appropriate care either as part of another study or using the local VA, military providers, and/or civilian resources as appropriate.

6.1.7. Compensation for participation.

Participants who are not active duty at the time of an assessment will receive $50 each time they complete comprehensive assessment measures at Baseline, 1, 3, or 6-month follow-up assessments for a possible total of $200. Participants will also be paid $25 each time they get their blood drawn (Baseline, twice during treatment, and 1-month and 6-months after treatment), for a possible total of $125. Additionally, participants will be paid $20 at each appointment during treatment to offset the cost of travel and potential lost income. Historically, the cost of traveling up to 12 times during treatment has been a barrier for some participants in the study. The possible total of $40 (2 sessions) to $240 (12 sessions) will help individuals who have long commutes, or may be hindered from lost work hours. Similarly, a payment of $0.50 will be given for each completed headache diary ($5 payment received with the milestone of 10 completed entries) for a possible total of $112. By completing all the diaries, the 4 assessments, up to 2-12 treatment sessions, and the 5 blood draws, participants could receive between $477 and $677 in total compensation. Participants who choose to withdraw from this study early will receive compensation for all assessments, treatment, diaries, and blood draws that they completed. Payment will be provided via a rechargeable MasterCard® ClinCard. The MasterCard® ClinCard is a debit card issued to the study participant. Funds are loaded onto card through the ClinCard website at www.clincard.com. Only authorized users will be able to access the ClinCard website to add funds with a username and password. The ClinCard funds will be available to recipients within 1 business day and can be used as the participant chooses. The participant will be notified that their name, address and date of birth will be shared with a third-party (ClinCard) solely for the purposes of payment processing. This information will only be used for the administration of the payment and will be kept strictly confidential. There will be no compensation for participation in the brief assessment of symptoms and discontinuation reasons for participants who discontinued participation in this study before completing treatment and follow-up assessments.

6.2. Study Procedures/Research Interventions.

Consent & Baseline Assessment

Individuals will visit the Audie L. Murphy VA Hospital (which is part of the South Texas Veterans Health Care System) or the UTHSCSA STRONG STAR clinic where they will learn more about the study, provide consent for participation. Baseline assessment will occur following consent. Most participants will be able to complete the baseline assessment at the same appointment, but individuals will be allowed to complete the baseline assessment appointment over two to three appointments if they prefer.

If participants are referred from other STRONG STAR studies, testing completed within the past one month as part of the assessment for other STRONG STAR studies will be used as baseline testing for participation in this study as appropriate with participant consent.

Participants found to meet inclusion/exclusion criteria will return to the clinic to have blood drawn as part of the baseline assessment, be oriented to the on-line daily diaries, and, if randomized to CPT-C, undergo a Trauma Interview conducted by the therapist who will be providing therapy. An appointment will be made for all participants approximately 2-weeks later to start treatment. The on-line headache diaries will be completed daily for approximately 2-weeks. If participants miss a headache diary entry, they will receive a phone call from research staff as needed to encourage and remind them to complete the diary.

Treatment

- Clinic-Based Cognitive Behavioral Therapy (CCBT). The CCBT headache treatment consists of 8 one-hour sessions that participants will be asked to complete in approximately 6 weeks (participants will be seen once or twice each week). Each treatment will take place at the South Texas Veterans Healthcare System (Audie Murphy Hospital) or the UTHSCSA STRONG STAR clinic. The 8 CCBT treatment sessions will cover many topics including: relaxation training, identifying stress, planning for long-term stress management, solving problems that impact their headaches, dealing with thoughts about their headache, specific headache coping skills training, and how to maintain their gains from treatment.
- Cognitive Processing Therapy-Cognitive Only (CPT-C). Cognitive Processing Therapy (CPT) is a trauma-focused treatment that involves learning to recognize and challenge thoughts related to traumatic brain

injury. Participants assigned to this treatment arm will be asked to meet with a study therapist twice a week for approximately 6-weeks for a total of 12 one-hour sessions. Each treatment will take place at the South Texas Veterans Healthcare System (Audie Murphy Hospital) or the UTHSCSA STRONG STAR clinic. During this treatment, participants will be asked to think about how they received their traumatic brain injury and their beliefs about the meaning of the event, as well as their current beliefs about themselves and others. Topics such as safety, trust, control, self-esteem, and intimacy will be discussed. Participants will be expected to complete assignments outside of session to practice skills learned in treatment.

- Treatment as Usual (TAU). Participants assigned into the Treatment as Usual (TAU) condition will receive treatment by their primary provider as indicated. The participants will meet with study staff twice during the treatment phase to complete assessments.

Assessments

- All participants regardless of treatment group will be asked to complete headache diary on-line twice a day.
- Blood will be drawn prior to treatment, during two of the treatment sessions, and at the 1- and 6-month assessments.
- Two weeks before the scheduled 1-, 3- and 6-month assessments, participants will once again be asked to complete headache diaries for two weeks prior to the evaluations.

All evaluations will be audio-recorded and CCBT and CPT-C therapy sessions will be audio-recorded to make sure that the study staff is correctly following the study procedures. These recordings will be reviewed by research experts who are part of the research team.

Assessment of Discontinued (i.e., treatment/assessment dropout) participants.

- All participants who discontinued participation in this study before completing study treatment and follow-up assessments will be contacted by phone by a project coordinator or research assistant and asked about their willingness to participate in a 10-15 assessment of their symptoms and reason for discontinuing participation. Participants will have the option to opt in to the assessment by giving verbal consent over the phone.
- Those who agree to participate will be asked questions about global change in headache and PTSD symptoms, their perceptions of study participation and study treatments, their reason(s) for discontinuing participation in the study, and verbal administration of the HIT-6 and PCL-5 to assess headache and PTSD symptom severity.

6.2.1 Collection of Human Biological Specimens.

All participants will have blood drawn prior to treatment, at two treatment sessions, and 1- and 6-months (following treatment completion) coordinated through the CAP Biomarkers and Genomics Core. A skilled phlebotomist will draw the samples. All blood samples will be collected at the Audie L. Murphy VA Hospital or the UTHSCSA STRONG STAR clinic.

6.2.1.1 Laboratory evaluations and special precautions.

All blood samples will be collected at the Audie L. Murphy VA Hospital or the UTHSCSA STRONG STAR clinic and then transported to the CAP Biomarkers and Genomics Core in the South Texas Research Facility (STRF) for processing and storage. This location is monitored 24-7 by a wireless monitoring system on the Isensix system and has approval by the STVHCS and Central VA office as a VA-Approved Tissue Bank. The study staff will be available to offer assistance if the participant is distressed at any point during any procedures.

6.2.1.2 Specimen storage.

Biological specimens will be coded with a digital bar code that can be linked to the study and subject only by key-codes maintained by the CAP Data Management and Biostatistics Core.

6.2.2 Data Collection.

Hardcopies of data will be stored in locked file cabinets within a dedicated, cipher/keyed room at the Audie L. Murphy VA Hospital in the Polytrauma Rehabilitation Center or the UTHSCSA STRONG STAR clinic (iaw local Privacy policies).

Electronic data will be stored on a FISMA compliant, secure, research database at UTHSCSA, https://delta.uthscsa.edu/ssdashboard/login.aspx. The STRONG STAR data server is physically located at the

Centralized Computing Facility (CCF) has 24x7 onsite security, card key access controls and video surveillance. University of Texas Health Science Center at San Antonio (UTHSCSA) CCF facility also maintains Gen 2 firewall devices to protect and prohibit any unauthorized access to UTHSCSA data. All UTHSCSA network devices are monitored by state of the art monitoring applications that include configuration audit, management, and availability 24x7.

Statistical Consideration

6.3.1 Sample Size Estimation.
This study will consent approximately 438 study participants with the goal of having 192 participants randomized for analysis.

6.3.2 Primary (i.e., primary outcome variables) and secondary endpoints.
The primary outcomes are headache symptoms and PTSD symptoms.

6.3.3 Data analysis.
Treatment groups will be compared on baseline variables. Dropout during treatment resulting in lack of outcome data can bias analyses. If attrition can be successfully modeled with logistic regression on the basis of baseline characteristics, and particularly if there is evidence of differential attrition, strategies such as inverse probability weighting can supplement the outcome analyses. Assuming that headache disability data (HIT-6; our primary outcome for this research) is reasonably normally distributed across the sample, treatment effects of the clinical interventions (CCBT and CPT-C) over the Treatment as Usual- Control condition will be analyzed using a general linear mixed (GLM) model with baseline scores used as a covariate. Unlike ANOVA, mixed effects models permit heterogeneity of variance in the treatment groups. If HIT-6 data are non-normal, a generalized linear model will be used (e.g., Poisson regression).

Two joint primary comparisons will be conducted comparing both active treatments to TAU. To adjust the alpha level for these joint hypotheses, we will interpret both at the alpha = 0.025 significance level. Secondary analyses will examine differences between the two active interventions as well as sensitivity analyses of the primary outcomes considering missing data, adherence to the protocol, or imbalances between the groups at baseline. Additional analyses will consider the secondary outcomes and differences between the groups at other time points.

A recent study helped to guide our determination of what constitutes a clinically meaningful change in perceived headache disability and the proposed study was powered in light of these differences. In the primary care setting, HIT-6 changes ranging from 2.5 (95% CI: -3.3 to -1.7) were perceived as "somewhat better" and changes up to 5.9 (95% CI: - 7.6 to -4.1) were perceived as "much better." We considered a meaningful change as somewhere between these two global impressions and used this marker as an index of clinically significant between-group differences. Our study is powered to detect an effect size of 2.8 points between groups after controlling for baseline scores (i.e., a residualized change) and constitutes an effect that would be meaningful to most headache sufferers. Assuming an alpha level of 0.025, group sample sizes of n = 64 (N = 192), and a moderate correlation between the baseline scores and final endpoints (r = 0.50), we will have power = 0.80 to detect an effect size of d = 0.52 between both of the joint primary comparisons. In realistic terms, this will allow us to detect a change of 2.8 points on the HIT-6 between the active treatment and the controls. Changes smaller than this magnitude are unlikely to be clinically meaningful.

6.4 Confidentiality.
Confidentiality will be safeguarded through a number of Consortium controls already in place for existing studies. First, all assessments will occur in a private room. Completed hard-copy assessments will be stored in a file in a locked drawer that is located in a locked office at the VA or the UTHSCSA STRONG STAR clinic. Patient identifiers are removed from all charts and associated only with a number identifier. Data will be transcribed into the Consortium database by the study coordinator or a trained research assistant using a double-entry method. All digital research data files will be encrypted and password- protected on the UT Health Science Center's FISMA-compliant network, with files accessible only to the Consortium Data Core. All UTHSCSA STRONG STAR network connectivity is segmented with Access Control Lists and is not accessible to any other UTHSCSA network segments. The STRONG STAR data server is physically located at the Centralized Computing Facility (CCF) has 24x7 onsite

security, card key access controls and video surveillance. University of Texas Health Science Center at San Antonio (UTHSCSA) CCF facility also maintains Gen 2 firewall devices to protect and prohibit any unauthorized access to UTHSCSA data. All UTHSCSA network devices are monitored by state of the art monitoring applications that include configuration audit, management, and availability 24x7. VA intervention and phone contacts will occur in the South Texas Veterans Health System at Audie Murphy VA Hospital in San Antonio, TX or the UTHSCSA STRONG STAR clinic. Dr. Carlos Jaramillo and Dr. Blessen Eapen have confirmed available office space for PTHA research.

Biological specimens will be coded with a digital bar code that can be traced back to the study and subject only by key-codes maintained by the Data Management and Biostatistics Core.

6.4.1 Certificate of Confidentiality. N/A

7.1 RISKS/BENEFITS ASSESSMENT

7.2 Risks.

Risks and side effects related to the Clinic-based Cognitive Behavioral Treatment (CCBT), Cognitive Processing Therapy- Cognitive Only (CPT-C) for post-traumatic headache, and blood draws include those which are:

Likely Risks, but Not Serious (expected to occur in less than 20 subjects out of 100),

- Psychological distress during assessments and treatment may produce some discomfort or emotional distress and can even produce a temporary increase in some symptoms in less than half of the people receiving the treatment. Similarly, treatment may be associated with a temporary or occasional increase in symptoms of depression, anxiety, or other pre-existing psychiatric symptoms.
- Discomfort, bruising or both, at the site of the needle puncture during blood sample collection.
- Some people experience fainting, the formation of a small blood clot or swelling of the vein and surrounding tissue, or bleeding from the puncture site.
- This research includes genetic research with the participant's samples. Using new technology, information about DNA structure (genetic information) can be used to indicate risk for developing certain diseases. This genetic information is unique to the participant and may indicate changes in their future health status or life expectancy, or that of their children and other relatives. If released, these discoveries could be stressful and cause psychological difficulties or family problems.

7.3 Potential Benefits.

The possible benefit of participating in this study is improvements in the severity and frequency of headaches and PTSD symptoms as well as a better understanding of headaches and posttraumatic stress.

8.0 ADVERSE EVENTS, UNANTICIPATED PROBLEMS, AND DEVIATIONS

The IRB policy on reporting adverse events, unanticipated problems, and protocol deviations will be followed.

9.1 WITHDRAWAL FROM STUDY PARTICIPATION.

Participants may be withdrawn from the study if:

- The researcher believes that it is not in their best interest to stay in the study.
- The participant becomes ineligible to participate.
- The participant's condition changes and they need treatment that is not allowed while taking part in the study.
- The participant does not follow instructions from the researchers.
- The study is stopped.

10.0 REFERENCES

1. Akshoomoff, N., Newman, E., Thompson, W. K., McCabe, C., Bloss, C. S., Chang, L., & Jernigan, T. L. (2014). The NIH Toolbox Cognition Battery: Results from a large normative developmental sample (PING). *Neuropsychology*, *28*(1), 1.
2. Antonaci, F., Nappi, G., Galli, F., Manzoni, G. C., Calabresi, P., & Costa, A. (2011). Migraine and psychiatric comorbidity: a review of clinical findings. *The journal of headache and pain*, *12*(2), 115-125.
3. Babor, T. F., Higgins-Biddle, J. C., Saunders, J. B. & Monteiro, M. G. (2001). *The Alcohol Use Disorders Identification Test: Guidelines for Use in Primary Care*, 2nd Edition. Geneva, Switzerland: World Health

Organization.

4. Bastien, C. H., Vallieres, A., Morin, C.M., (2001) Validation of the Insomnia Severity Index as an outcome measure for insomnia research. *Sleep Medicine, 2*(4), 297-307.
5. Beck, A. T., Epstein, N., Brown, G. & Steer, R. A. (1988). An inventory for measuring clinical anxiety: Psychometric properties. *Journal of Consulting and Clinical Psychology, 56*, 893-897.
6. Beck, A. T. & Steer, R. A. (1991). *Manual for the Beck Scale for Suicide Ideation.* San Antonio, TX: The Psychological Corporation.
7. Beck, A. T., Steer, R. A., & Brown, G. K. (1996). *Manual for the BDI-II*. San Antonio, TX: The Psychological Corporation.
8. Beck, A. T., Ward, C. H., Mendelson, M., Mock, J. & Erbaugh, J. (1961). An inventory for measuring depression. *Archives of General Psychiatry, 4*, 564-571.
9. Brantley, P. J., Waggoner, C. D., Jones, G. N., & Rappaport, N. B. (1987). A daily stress inventory: Development, reliability, and validity. *Journal of behavioral medicine*, *10*(1), 61-73.
10. Breslau, N., Lipton, R. B., Stewart, W. F., Schultz, L. R., & Welch, K. M. A. (2003). Comorbidity of migraine and depression investigating potential etiology and prognosis. *Neurology*, *60*(8), 1308-1312.
11. Borkovec, T. D & Nau, S. D. (1972). Credibility of analogue therapy rationales. *Journal of Behaviour Therapy and Experimental Psychiatry, 3*, 257-260.
12. Campbell, J. K., Penzien, D. B., Wall, E. M. (2000). Evidence-based guidelines for migraine headaches: Behavioral and physical treatments. American Academy of Neurology Web site (http://www.aan.com/professionals/practice/pdfs/g10089.pdf). Accessed Jan. 25, 2012.
13. Caplan, L. J., Ivins, B., Poole, J. H., Vanderploeg, R. D., Jaffee, M. S., & Schwab, K. (2010). The structure of postconcussive symptoms in 3 US military samples. *The Journal of Head Trauma Rehabilitation*, *25*(6), 447-458.
14. Cella, D., Riley, W., Stone, A., Rothrock, N., Reeve, B., Yount, S., ... & Hays, R. (2010). The Patient-Reported Outcomes Measurement Information System (PROMIS) developed and tested its first wave of adult self-reported health outcome item banks: 2005–2008. *Journal of clinical epidemiology*, *63*(11), 1179-1194.
15. Chervin, R.D., Aldrich, M.S., Pickett, R., & Guilleminault, C. (1997). Comparison of the results of the Epworth Sleepiness Scale and the Multiple Sleep Latency Test. *Journal of Psychosomatic Research, 42*(2),145-155.
16. Chung, F., Yegneswaran, B., Liao, P., Chung, S. A., Vairavanathan, S., Islam, S., Khajehdehi, A., & Shapiro, C. M. (2008). STOP questionnaire: A tool to screen patients for obstructive sleep apnea. *Anesthesiology, 108*, 812-921.
17. Corrigan, J.D., & Bogner, J.A. (2007). Initial reliability and validity of the OSU TBI Identification Method. *J Head Trauma Rehabil, 22***(**6):318-329.
18. Davis, J. L., & Wright, D. C. (2007). Randomized clinical trial for treatment of chronic nightmares in trauma-exposed adults. *Journal of Traumatic Stress*, *20*(2), 123-133.
19. Davis, J. L., Wright, D. C., & Borntrager, C. (2001). *The Trauma-Related Nightmare Scale*. Tulsa, OK: University of Tulsa, Department of Psychology.
20. Devilly, G. J., & Borkovec, T. D. (2000). Psychometric properties of the credibility/expectancy questionnaire. *Journal of Behavior Therapy and Experimental Psychiatry, 31*, 73-86.
21. Foa, E. B., Elhers, A., Clark, D. M., Tolin, D. F. & Orsillo, S. M. (1999). The Posttraumatic Cognitions Inventory (PCTI). *Psychological Assessments, 11*, 303-314.
22. French, D. J., Holroyd, K. A., Pinell, C., Malinoski, P. T., O'donnell, F., & Hill, K. R. (2000). Perceived Self-efficacy and Headache-Related Disability. *Headache: the journal of head and face pain*, *40*(8), 647-656.
23. Garnefski, N. & Kraaij, V. (2006). Cognitive emotion regulation questionnaire – development of a short 18-item version (CERQ-short). *Personality and Individual Differences, 41*, 1045-1053.
24. Garnefski, N., Kraaij, V. & Spinhoven, P. (2001). Negative life events, cognitive emotion regulation and emotional problems. *Personality and Individual Differences, 30*, 1311-1327.
25. Goslin RE, Gray RN, McCrory DC, Penzien D, Rains J, Hasselblad V. Behavioral and Physical Treatments for Migraine Headache (Technical Reviews, No. 2.2). Rockville (MD): Agency for Health Care Policy and Research (US); 1999 Feb. (http://www.ncbi.nlm.nih.gov/books/NBK45267/?part=). Accessed Oct. 6, 2011.
26. Gray, M., Litz, B., Hsu, J., & Lombardo, T. (2004). Psychometric properties of the Life Events Checklist. (PDF) Assessment, 11, 330-341. doi: 10.1177/1073191104269954

27. Harris, P. A., Taylor, R., Thielke, R., Payne, J., Gonzalez, N., & Conde, J. G. (2009). Research electronic data capture (REDCap)- A metadata-driven methodology and workflow process for providing translational research informatics support. *J Biomed Inform, 42(2),* 377-381.
28. Jeffery, D. D., Tzeng, J. P., Keefe, F. J., Porter, L. S., Hahn, E. A., Flynn, K. E., ... & Weinfurt, K. P. (2009). Initialreport of the cancer Patient-Reported Outcomes Measurement Information System (PROMIS) sexual function committee. *Cancer*, *115*(6), 1142-1153.
29. Johns, M.W. (1994). Sleepiness in different situations measured by the Epworth Sleepiness Scale. *Sleep.17*(8),703- 710.
30. Johnson, D. C., Polusny, M. A., Erbes, C. R., King, D., King, L., Litz, B. T., Schnurr, P. P., Friedman, M., Pietrzak, R. H. & Southwick, S. M. (2008). Resilience and response to stress: Development and initial validation of the Response to Stressful Experiences Scale (RSES). Unpublished manuscript, Naval Health Research Center, San Diego, CA.
31. Jones, D., Kazis, L., Lee, A., Rogers, W., Skinner, K., Cassar, L., et al. (2001). Health status assessments using the Veterans SF-12 and SF-36: Methods for evaluating outcomes in the Veterans Health Administration. *The Journal of Ambulatory Care Management*, *24*, 68–86.
32. Kazis LE, Lee A, Spiro III. A, Rogers W, Ren XS, Miller DR, Selim A, Hamed A, and Haffer SC. (2004). Measurement comparisons of the Medical Outcomes Study and the Veterans SF-36® Health Survey. *Health Care Financing Review, 25*(4), 43-58.
33. Kazis LE, Selim A, Rogers W, Ren XS, Lee A and Miller DR. (2006). Dissemination of methods and results from the Veterans Health Study: Final comments and implications for future monitoring strategies within and outside the Veterans Health Care System. *Journal of Ambulatory Care Management, 29(*4), 310-319.
34. Kosinski MS, Bayliss JB, Bjorner JE, et al. A six-item short-form survey for measuring headache impact: The HIT-6™. Quality of Life Research. 2003;12:963-974.
35. Kroenke, K., Spitzer, R. L., & Williams, J. B. (2002). The PHQ-15: Validity of a new measure for evaluating the severity of somatic symptoms. *Psychosomatic medicine*, 64, 258-266.
36. Kubany, E. S., Haynes, S. N., Abueg, F. R., Manke, F. P., Brennan, J.M., Stahura, C. (1996). Development and validation of the Trauma-Related Guilt Inventory (TRGI). *Psychological Assessments, 8*, 423-444.
37. Lichstein, K. L., Durrence, H. H., Riedel, B. W., Taylor, D. J., & Bush, A. J. (2004). *Epidemiology of sleep: Age, gender, and ethnicity*. Psychology Press.
38. Martin, N. J., Holroyd, K. A., & Penzien, D. B. (1990). The Headache-Specific Locus of Control Scale: Adaptation to Recurrent Headaches. *Headache: the journal of head and face pain*, *30*(11), 729-734.
39. Monteith, T., Boylan, L., & Young, W. B. (2009).Teaching case presentation: heroic headache. In: Levin M, ed. Resident and fellow section. *Headache, 49(2),* 325-327. doi:10.1111/j.1526-4610.2008.01331.x
40. Morin, C.M. (1993). Insomnia: Psychological Assessment and Management. New York, NY: The Guilford Press.
41. Posner, K., Oquendo, M. A., Gould, M., Stanley, B., & Davies, M. (2007). Columbia Classification Algorithm of Suicide Assessment (C-CASA): Classification of suicidal events in the FDA's pediatric suicidal risk analysis of antidepressants. *American Journal of Psychiatry, 164,* 1035–1043.
42. Resick, P. A., Falsetti, S. A., Resnick, H. S., & Kilpatrick, D. G. (1991). The Modified PTSD Symptom Scale—Self Report. *Charleston, SC, Crime Victims Treatment and Research Center, Medical University of South Carolina*.
43. Ruff, R. L., Riechers, R. G., Wang, X. F., Piero, T., & Ruff, S. S. (2012). A case–control study examining whether neurological deficits and PTSD in combat veterans are related to episodes of mild TBI. *BMJ open*, *2*(2).
44. Saunders, J. B., Aasland, O. G., Babor, T. F., De La Fuente, J. R. & Grant, M. (1993). Development of the Alcohol Use Disorders Identification Test (AUDIT): WHO collaborative project on early detection of persons with harmful alcohol consumption-II. *Addiction, 88*, 791-804.
45. Shacham, S. (1983). A shortened version of the Profile of Mood States. *Journal of personality assessment*, *47*(3), 305-306.
46. Shipherd, J. C., Clum, G., Suvak, M., & Resick, P. A. (2013). Treatment-related reductions in PTSD and changes in physical health symptoms in women. *Journal of behavioral medicine*, 1-11.
47. Schwedt TJ, Dodick W, Hentz J, et al. Occipital nerve stimulation for chronic headache: Long-term safety and efficacy. Cephalgia. 2007;27:153-157.
48. Silberstein, S. D. (2000). Practice parameter: evidence-based guidelines for migraine headache (an evidence-based review): report of the Quality Standards Subcommittee to the American Academy of Neurology. *Neurology, 55(6),* 754-762.

49. Steer, R.A., Rissmiller, D.J., Ranieri, W.F., Beck, A.T. (1993). Dimensions of suicidal ideation in psychiatric inpatients.
Behaviour Research and Therapy, 31(2), 229-236.
50. Vogt, D., Smith, B. N., King, D. W., & King, L. A. (2012). *Deployment Risk and Resilience Inventory-2 (DRRI-2).* Assessment available from the National Center for PTSD at www.ptsd.va.gov.
51. Watanabe, T. K., Bell, K. R., Walker, W. C., & Schomer, K. (2012). Systematic review of interventions for post-traumatic headache. *PM&R*, *4*(2), 129-140.
52. Weathers, F.W., Blake, D.D., Schnurr, P.P., Kaloupek, D.G., Marx, B.P., & Keane, T.M. (2013a). *Life Events Checklist-5(LEC-5)*. Assessment available from the National Center for PTSD at www.ptsd.va.gov.
53. Weathers, F.W., Blake, D.D., Schnurr, P.P., Kaloupek, D.G., Marx, B.P., & Keane, T.M. (2013b). *The Clinician-Administered PTSD Scale for DSM-5 (CAPS-5)*. Interview available from the National Center for PTSD at www.ptsd.va.gov.
54. Weathers, F. W., Litz, B. T., Keane, T. M., Palmieri, P. A., Marx, B. P., & Schnurr, P. P. (2010). The PTSD Checklist for DSM–5 (PCL-5).

Description of Study Measures

1. Ohio State University Traumatic Brain Injury Identification Short Form (OSU TBI-ID-SF): The OSU TBI-ID-SF is a 6 item screener used to determine the likelihood an individual suffered a TBI. Those showing a history of probably TBI will be included in the study (Corrigan & Bogner, 2007).
2. History of Head Injury: We will use a modified version of the Defense and Veterans Brain Injury Center (DVBIC) 3 Item Screening Tool (Schwab, Baker, Ivins, Sluss-Tiller, Lux & Warden, 2006; Schwab, Ivins, Cramer, Johnson, Sluss-Tiller, Kiley, Lux & Warden, 2006) that was used in STRONG STAR. This instrument, initially called the Brief Traumatic Brain Injury Screen (BTBIS), was used as the gold standard for the diagnosis of TBI in a sample of soldiers returning from duty in Iraq and/or Afghanistan (Schwab, Ivins, et al., 2006). As recommended by the DVBIC, the 3- Question Screen will be considered positive when the participant endorses an injury (question 1) and altered consciousness (question 2, items A-E) for the worst head injury sustained while deployed. The form was modified for STRONG STAR and now CAP to capture the number of injuries, and to answer question 2 based on the worst injury; the original form does not recognize the possibility of multiple head injuries during deployment. As the 3-Question Screen does not query head injuries prior to deployment, an additional four questions have been added to solicit information about each head injury sustained outside of deployment.
3. Response to Stressful Experiences Scale (RSES) (previously known as the National Center for PTSD Trait Resilience Scale). The RSES is a 22-item questionnaire developed by a team of experts at the National Center for PTSD to assess trait-related cognitive, emotional, and behavioral resilience (Johnson, et al., 2008). It asks participants to assess how well each statement describes them, both during and after stressful events in their lives. Responses are given on a 5-point scale, with anchors 0 (not at all like me) to 4 (exactly like me). Psychometric testing in 1,014 active duty, reserve and veteran groups showed that the instrument has sound internal consistency (coefficient alpha 0.91 to 0.93) as well as good test-retest reliability over 7-days (reliability correlation = 0.87). The instrument correlated positively with another measure of resilience, the Connor-Davidson Resilience Scale (coefficient alpha 0.61 to 0.81) as well as unit cohesion (coefficient = 0.38), and post-deployment support (coefficient 0.36 to 0.56). The instrument correlated negatively with psychological symptom distress as assessed with the Patient Health Questionnaire - 9 (coefficient = -0.51), posttraumatic stress as assessed with the PCL-M (coefficient -0.23 to -0.39), and overall mental health as assessed with the Minnesota Multiphasic Personality Inventory-2 Neuroticism (coefficient = -0.35) demonstrating concurrent validity. Factor analysis revealed a six-factor model of resilience including subscales for active coping, meaning-making, cognitive flexibility, spirituality, self-efficacy, and restoration.
4. Cognitive Emotions Regulation Questionnaire (CERQ)-Short. The CERQ was designed to assess cognitive coping strategies people tend to use, or what someone thinks, after having experienced threatening or stressful events (Garnefski, Kraaij, & Spinhoven, 2001). The CERQ can be used to assess cognitive strategies that characterize the individual's style of responding to stressful events. Thirty six (36)-items are scored to produce nine subscales including: 1) Self-Blame, 2) Blaming Others, 3) Rumination, 4) Catastrophizing, 5) Positive Refocusing, 6) Planning, 7) Positive Reappraisal, 8) Putting into Perspective, and 9) Acceptance. The answer categories for each item range from 1 (almost never) to 5 (almost always). Individual subscale scores are obtained by summing up the scores belonging to the particular subscale. The

higher the subscale score, the more a specific cognitive strategy is used. Psychometric testing of the 36-item CERQ was conducted using a group of 547 secondary school students aged 12 to 16 attending a state school in the Netherlands. Internal consistency of the subscales ranged from 0.71 to 0.92. The CERQ was positively correlated with both depression and anxiety as assessed with the subscales of the Symptom Check List-90. Test-retest

reliability for each of the subscales at five months ranged from 0.41 to 0.59. The CERQ-short form (Garnefski & Kraaij, 2006) is an 18-item questionnaire that produces the same 9 subscales as the 36-item questionnaire. A study of 611 adults from a general practitioner's office practice in the Netherlands indicated that the internal consistency of the 2-item subscales remained acceptably high (α = 0.67 to 0.81). In support of the validity of the CERQ-short, correlations with outcome measures were comparable to reported results with the original CERQ in that the Rumination, Self-Blame and Catastrophizing subscales were related to more depression and anxiety symptoms, while the Positive Reappraisal subscale was related to fewer symptoms (Garnefski & Kraajj, 2006).

5. Demographics: We will use a standard CAP form to gather relevant demographics including: age, gender, ethnicity, date of concussion/TBI, mechanism of concussion/TBI, number of past concussions/TBIs, relevant comorbid medical conditions (e.g., cognitive decline, musculoskeletal or neuropathic pain –especially presenting in the head or neck).
6. Health Interview (Pre-Treatment) and Health Interview (Follow-Up) also, pre- and post- HI addendum: The Health Interview includes items regarding general health, hospitalizations, current and past psychiatric medications, thoughts about wanting to harm others, utilization of mental health services, utilization of outpatient medical services, and caffeine and tobacco use. Additionally, the health interview will include a review of the participant's medical record to identify provider-assigned ICD-9 diagnostic codes for chronic and neuropathic pain.
7. Generalized Anxiety Disorder Screener (GAD-7): The GAD-7 (Spitzer, Kroenke, Williams, & Lowe, 2006) will be used to assess generalized anxiety symptomology. This is a 7-item measure that asks participants to rate the frequency with which they have been bothered by anxiety symptoms within the past two weeks on a scale ranging from 0 ("not at all") to 3 ("nearly every day"). Scores on all items are summed to obtain a total severity score. Scores reflect no significant anxiety symptoms (0-4), mild anxiety symptoms (5-9), moderate anxiety symptoms (10-14), and severe anxiety symptoms (>15). Respondents also indicate the degree to which their anxious symptoms have made it difficult for them to do their work, take care of things at home, or get along with other people, from "not difficult at all" to "extremely difficult." The GAD-7 has been shown to have high internal consistency (e.g., α = .89; Lowe et al., 2008) and has been shown to reliably discriminate between anxious and non-anxious diagnostic groups (Kroenke, Spitzer, Williams, & Lowe, 2010).
8. Self-Injurious Thoughts and Behaviors Interview (SITBI): The Self-Injurious Thoughts and Behaviors Interview (SITBI; Nock, Holmberg, Photos, & Michel, 2007) is a structured interview assessing the presence, frequency, and characteristics of self-injurious and suicidal thoughts and behaviors. The SITBI will be administered by an Independent Evaluator, who will instruct the participants to answer the questions based on their entire lifetime of experience. The SITBI has shown high interrater reliability, test-retest reliability, and concurrent validity (Nock et al., 2007).
9. Deployment Risk and Resilience Inventory (DRRI - II) Combat Experience, Postbattle Experiences, and Deployment Environment Sub-Scales: High- and low-intensity deployment stress exposure will be assessed using scales from the DRRI-2 (Vogt, Smith, King, & King, 2012). High intensity stressor exposures will be assessed using the DRRI-2 *Combat Experiences* and *Postbattle Experiences* subscales. Responses to these scales are on a 6-point Likert scale. The total score is the sum of the item scores, where higher scores signify greater exposure to combat or exposure to the consequences of combat, respectively. In addition, low-intensity deployment stress will be assessed with the DRRI-2 *Deployment Environment* subscale. Responses to this scale are on a 5-point Likert scale with anchors 1 (*almost none of the time*) to 5 (*almost all of the time*). The total score equals the sum of the item scores, where higher scores are indicative of a more difficult living and working environment
10. Life Events Checklist (LEC-5): The LEC-5 (Weathers, Blake, Schnurr, Kaloupek, Marx, & Keane, 2013) includes the same list of 16 different potentially traumatic life events from the original LEC that are commonly associated with PTSD symptoms and designed to facilitate PTSD diagnosis (Gray, Litz, Hsu, & Lombardo, 2004). There is also a blank for specifying an additional stressful event not encompassed in the 16 events. For each potentially traumatic life event, respondents rate their experience of that event on a 6-point nominal scale (1 = happened to me, 2 = witnessed it, 3 = learned about it, 4 = part of my job, 5 = not sure, and 6 = doesn't apply). The primary addition to the LEC-5 is a category involving occupational exposure ("for example, paramedic, police, military,

or other first responder"). There has not been a publication on the psychometric properties of the LEC-5, but the measure is nearly identical to the original LEC. In a group of 108 undergraduate psychology students the LEC demonstrated good convergence with the Traumatic Life Events Questionnaire (average kappa = 0.55) and correlated with the Posttraumatic Stress Disorder Checklist – Civilian version (reliability coefficients 0.34 to 0.48). The LEC demonstrated good test-retest reliability over 7 days. In 131 combat veterans the LEC was related in the predicted directions with other measures of psychopathology known to be associated with potentially traumatic life events as assessed by the Posttraumatic Stress Disorder Checklist – Military version, Clinician-Administered PTSD Scale, and the Mississippi Scale for Combat-Related PTSD.

11. <u>Posttraumatic Cognitions Inventory (PTCI). The PTCI is a 36-item questionnaire that was developed to deter</u>mine how an individual views the trauma and its sequelae in an attempt to understand both how PTSD develops and is maintained (Foa, Elhers, Clark, Tolin, & Orsillo, 1999). Using an emotional processing theory, Foa and her colleagues (1999) have suggested that PTSD is a consequence of disruptions in the normal processes of recovery when an individual has excessively rigid concepts about self and world rendering the person vulnerable if a traumatic event occurs. Thus the PTCI was developed as a measure of trauma-related thoughts and beliefs. It is comprised of three subscales (Negative Cognitions about the Self, Negative Cognitions about the World, and Self-Blame). The measure was tested in almost 600 adult volunteers recruited from two university PTSD treatment clinics as well as a university community. Approximately 65% (n=392) of individuals reported having experienced a trauma in which their own life or that of another person was perceived to be in danger and their response at the time included intense terror, horror, or helplessness (Criterion A event). The remaining 35% (n=162) denied such a traumatic experience. Of those who had experienced a trauma, 170 had PTSD symptoms of at least moderate severity while the remaining 185 reported a low symptom severity. The three subscales of the PTCI demonstrated internal consistency with alpha coefficients ranging from 0.86 to 0.97. Convergent validity was demonstrated comparing the PTCI to appropriate subscales of the World Assumptions Scale and Personal Beliefs and Reactions Scale. Significant correlations between the appropriate subscales ranged from 0.20 to 0.85. The PTCI was able to differentiate individuals with and without PTSD demonstrating discriminate validity (sensitivity = 0.78, specificity = 0.93). Test–retest reliability for each of the three subscales at a 1-week interval ranged from 0.75 to 0.89 and for a 3-week interval ranged from 0.80 to 0.86.
12. <u>Structured Diagnostic Headache Interview-Revised (Brief Version; SDIH-R):</u> The SDIH-R is a structured clinical interview assessing headache symptoms that conform to the ICHD-II criteria for primary and posttraumatic headache (Silberstein, 2000; Campbell, Penzien, & Wall, 2000). This interview will be administered at the baseline assessment session to further establish the participant's headache diagnosis and again at post-treatment and six-month follow-ups (to help establish the perseverance or dissolution of the PTHA diagnosis in response to treatment.
13. <u>Daily Headache Diary (Online)</u> (Goslin et al, 1999): Headache frequency and severity will be assessed via daily headache self-monitoring diaries. Patients will complete the assessment twice daily (during AM and PM), requiring 5 to 10 minutes per assessment. Although the descriptions of when the various forms are completed are made in reference to a typical sleep-wake cycle, it is of note that the "morning" (AM) entry simply refers to the entry that is made when a subject awakens and the "evening" (PM) entry is made at the end of their waking day (thus, the entries can easily be adapted for individuals doing shift work). They will record their headache intensity 2 times during the day using a 0 to 10 scale, with 0 representing "no headache" and 10 representing a headache that was "extremely painful—I can't do anything when I have this headache." From these diaries, 4 dependent measures will be computed for each assessed week: (a) days per week with a headache (days with headache greater than 2); (b) weekly headache frequency (total number of discrete headaches per week); (c) headache duration (total number of hours of headache per week with headache greater than 2); and (d) average peak headache intensity. The Headache Diary also tracks the occurrence of known headache triggers and medication use for headache management (prophylactic and abortive).
14. <u>Headache Impact Test-6 (HIT-6)</u>: The HIT-6 is a 6-item measure of headache-disability considered a gold standard in headache clinical and research work. Developed in 2003 (Kosinski et al, 2003), the HIT-6 has demonstrated excellent reliability (internal consistency = 0.89; test-retest reliability = 0.80) and validity (relative validity coefficients ranging from 0.82 to 1.00). There are almost 100 published studies of headache using the HIT-6, including previous studies of PTHA (Schwedt et al, 2007).
15. <u>Headache Management Self-Efficacy Scale (HMSE)</u>: This is a 25-item assessment rated on a 7 point Likert-type scale measuring one's confidence at preventing headaches (French, Holyroyd, & Peinell, et al, 1999).

16. Headache Specific Locus of Control Scale (HSLC): The HSCL is a 33 item measures scored on a 5-point Likert–type scale to measure an individual's perceptions that headache problems and relief are determined by: individual behaviors, healthcare professionals, or chance factors (Martin, Holyroyd, & Penzien, 1990).
17. Clinician-Administered PTSD Scale for DSM-5 (CAPS-5) : The CAPS-5 is structured interview that assesses the DSM-5 criteria for PTSD (Weathers et al., 2013). Each item is rated on a severity scale ranging from 0 (Absent) to 4 (Extreme/incapacitating) and combines information about frequency and intensity for each of the 20 symptoms. Additional items that are not included in the total score evaluate overall symptom duration, distress, impairment, dissociative symptoms, and global ratings by the interviewer. Validation studies are nearly complete to establish the psychometric properties of the CAPS-5 and findings will be reported in peer-reviewed publications. This interview is very similar to its predecessor, the CAPS for DSM-IV, which has been considered the gold standard for evaluating PTSD and demonstrated good reliability and validity (Weathers, Keane, & Davidson, 2001). In addition to reflecting diagnostic changes for PTSD in DSM-5, the CAPS-5 differs from the CAPS in that frequency and intensity ratings for each symptom are no longer scored separately, so the severity rating for each item determines whether a symptom is present or not. Subscale scores are calculated by summing severity scores for items in the following PTSD symptom clusters: re-experiencing, avoidance, negative alterations in cognitions and mood, and hyperarousal.
18. PTSD Checklist –DSM-5 (PCL-5): The PCL-5 (Weathers, et al., 2010) is a 20-item self-report measure update of the PCL designed to assess PSTSD symptoms as defined by the DSM-5. The PCL-5 is currently available and has been shown to have good psychometric properties.
19. Neurobehavioral Symptom Inventory (NSI): The NSI is a 21-item assessment of post-concussive symptoms with a 3- factor structure (somatic/sensory, affective, and cognitive) (Caplan et. al, 2010).
20. Patient Health Questionnaire-9 (PHQ-9): The PHQ-9 is a widely used and well-validated instrument for measuring the severity of depressive symptoms (Kroenke, Spitzer, & Williams, 2001). It consists of 9 items that assess both affective and somatic symptoms related to depression and depressive disorders; these 9 items correspond to the diagnostic criteria for DSM MDD. Respondents rate the frequency with which they have been bothered by depressive symptoms on a scale ranging from 0 ("not at all") to 3 ("nearly every day"). Scores on all items are summed to obtain a total severity score. Scores reflect no significant depressive symptoms (0-4), mild depressive symptoms (5-9), moderate depressive symptoms (10-14), moderately severe depressive symptoms (15-19), and severe depressive symptoms (>19). Respondents also indicate the degree to which their depressive symptoms have made it difficult for them to do their work, take care of things at home, or get along with other people, from "not difficult at all" to "extremely difficult." The PHQ-9 has high internal consistency (e.g., alpha ranging from .83 to .92; Cameron, Crawford, Lawton, & Reid, 2008), and correlates strongly with other measures of depression
(Kroenke et al., 2001).
21. Online Profile of Mood States-Short Form (POMS-SF; Shacham, 1983) online Each morning and evening, subjects will complete the POMS-SF. The POMS-SF is a 37-item scale which assesses distinct mood states. Each emotional adjective (e.g., "sad") is rated on a 0 ("Not at all") to 4 ("extremely") scale. The measure takes approximately 1.5 – 3.5 minutes to complete.
22. Online Daily Stress Inventory (DSI; Brantley et al., 1987) online Each evening subjects will complete the DSI. The DSI is a 58-item scale that allows a subject to identify stressful events that they have experienced in the last 24 hours. For each item that is endorsed, a stressfulness impact rating is made ranging from 1 ("occurred but was not stressful") to 7 ("caused me to panic").
23. Depressive Symptom Index – Suicidality Subscale (DSI-SS): The DSI-SS (Metalsky & Joiner, 1997) will be used to assess current suicidal ideation. The DSI-SS is a 4-item self-report measure of suicidal ideation that focuses on ideation, plans, perceived control over ideation, and impulses for suicide. It is being used as a core measure in the Military Suicide Research Consortium. Scores on each item range from 0 to 3, with higher scores reflecting greater severity of suicidal ideation. Instructions will instruct the participants to respond based on the past week. A systematic review of measures of suicidal ideation and behaviors found that the DSI-SS had evidence of excellent internal consistency and concurrent validity (Batterham et al., 2014).
24. NIH Toolbox Cognition Battery: The NIH Toolbox is a recently developed comprehensive assessment tool with an emphasis on measuring outcomes in longitudinal epidemiologic studies and prevention or intervention trials across a lifespan (Akshoomoff, et al., 2014). The cognition battery is a brief and efficient computer-based neuropsychological test of the seven key cognitive domains: Executive Functioning, Episodic Memory, Working Memory, Processing Speed, Language, Attention and Reading. The oral reading subtest will not be administered to reduce subject burden. The battery requires only 20-30 minutes to administer.

25. Alcohol Use Disorders Identification Test (AUDIT) self-report version: The AUDIT (Babor et al., 2001) will be used to identify people with hazardous or harmful patterns of alcohol consumption and to index the severity of these problems. It will be administered as a self-report form. The AUDIT is a 10-item screening measure, developed by the World Health Organization (WHO), with three subscales (alcohol consumption, drinking behavior, and alcohol-related problems) that are scored on a 4-point scale for a highest possible total score of 40. The AUDIT has good internal consistency (αs = .80-.93) as well as sensitivity and specificity (Saunders, Aasland, Babor, De La Fuente & Grant, 1993; see Reinert & Allen, 2007, for review).
26. Quick Drinking Screen (QDS) self-report version: The QDS (Sobell et al., 2003) will be used to measure alcohol consumption. It consists of 4 items probing frequency and quantity of alcohol consumption. It will be administered in a self-report form. The QDS has been validated against the Timeline Followback daily estimation measure of alcohol use, and it shows good psychometric properties (Roy et al., 2008; Sobell et al., 2003). The QDS's time-frame will be modified to match the "last two weeks" probed by the mandated depression and anxiety instruments for CAP studies (PHQ-9 and GAD-7). Like these other measures, the QDS can be administered frequently throughout CAP trials to track changes in alcohol use.
27. Brief Inventory of Psychosocial Functioning (B-IPF): This is a 7-item self-report instrument measuring respondents' level of functioning in seven life domains: romantic relationship, relationship with children, family relationships, friendships and socializing, work, training and education, and activities of daily living (Marx, 2013). Respondents indicate the degree to which they had trouble in the last 30 days in each area on a 7-point scale ranging from "0 = Not at all" to "6 = Very much." The B-IPF has demonstrated concurrent validity, and the full 80-item IPF from which it was created has strong test-retest reliability and internal consistency (Marx, 2013).
28. Patient Health Questionnaire – 15 (PHQ-15): The PHQ-15 (Kroenke, Spitzer, & Williams, 2002) is an abbreviated version of the original PHQ that asks about somatic symptoms and symptom clusters that account for more than 90% of physical complaints reported in an outpatient setting. The 15-item measure asks patients to report symptom severity on a scale ranging from 0 ("not bothered at all") to 2 ("bothered a lot"). The PHQ-15 has been found to have excellent internal reliability (α = .80) and good convergent validity with scales such as the SF-12 and other measures of symptom severity and functionality (Kroenke, Spitzer, & Williams, 2002).
29. Veterans Rand 12-Item Health Survey (VR-12): The Veterans SF-12 was developed from the Veterans SF-36 (VR-36) and adapted from the MOS SF-36, and spans the range of health domains from physical to psychological health status. The VR-36 has been widely used, distributed and documented in the Veterans Health Administration (VHA). The VR-36 assesses eight domains including physical functioning, role limitations due to physical problems, bodily pain, general health perceptions, vitality, social functioning, role limitations due to emotional problems and mental health. The eight scales are summarized into two summary scores, physical (PCS) and mental (MCS). Higher scores indicate better health. The VR-12 includes 12 items representing the eight domains from the VR-36 and two items measuring change in physical and mental health. The VR-12 explains 90% of the reliable variance of the VR-36 (Jones, et al., 2001). Using independent results from the Veterans Health Study and the 1996 National Survey of Ambulatory Care Patients, the results for the Veterans SF-12 corresponded very closely with the results for the Veterans SF-36 (Kazis, et al., 2004; Kazis, et al., 2006).
30. Online Daily Diary Sleep Quantity/Quality (Lichstein et. al, 2004) online Each morning (once per day on awakening), subjects will record the following in the electronic diary: A) What time did you get out of bed this morning? B) What time did you go to bed last night? C) How long did it take for you to fall asleep after going to bed last night? D) How many times did you wake up during the night? E) How long were you awake during these awakenings in the night (in total)? F) About how long did you sleep during the night (altogether)? G) How would you rate the quality of your sleep? [0-4 scale; 0=poor; 2=fair; 4=excellent] H) How well (rested and refreshed) do you feel this morning? [0-4 scale; 0=not at all; 2=moderately; 4=very] I) Did you nap or doze yesterday before going to bed? (If so, what time and how long did you sleep)?
31. Patient Reported Outcomes Measurement Information System (PROMIS) Sleep Disturbance and Sleep-Related Impairment short forms: The PROMIS Sleep Disturbance and Sleep-Related Impairment short forms (Yu, Buysse, & Germain, 2012) are self-report measures of past-week sleep disturbance and past-week sleep-related impairment, respectively, derived from the larger PROMIS item banks (Buysse et al., 2010). Each short-form measure includes 8 items, with most items (symptoms) scored in intensity from 1 ("not at all") to 5 "(very much"). Each measure has shown strong reliability and construct validity (Yu et al., 2012).
32. Snoring, Tired, Observed, Blood Pressure (STOP) Sleep Apnea Screen (Chung et al., 2008). To better understand sleep disturbance associated with PTSD and PTSD treatment, the STOP screen will be

administered to screen for sleep apnea. The STOP is a four-item questionnaire developed and validated in 211 pre-operative surgical patients. Using the answering of 2 or more questions "yes," the sensitivity of the STOP ranged from 66% to 80% as compared with the apnea-hypopnea index (AHI) of polysomnography depending upon the AHI cut-off used. Individuals answering "yes" to 2 or more of the questions will be advised that they may be at risk for having sleep apnea and advised that they may want to speak with their primary care provider to consider referral for an overnight sleep evaluation at the CRDAMC Sleep Clinic

33. Insomnia Severity Index (ISI; Morin, 1993). The ISI is a 7-item self-report measure that assesses perceived severity of insomnia. Each item uses a 4-point Likert type scale from 0 (not at all satisfied) to 4 (very much satisfied). The items sum to produce a total score (range 0 – 28). The ISI has an internal consistency alpha coefficient of 0.74, and has shown convergent validity with other measures such as the Pittsburgh Sleep Quality Index (r = 0.67), the Dysfunctional Beliefs and Attitudes about Sleep (r = 0.55), and sleep diaries (range from 0.32-0.91) (Bastien, Vallieres & Morin, 2001).
34. Patient Reported Outcomes Measurement Information System (PROMIS) Sexual Functioning Profile: The PROMIS questionnaires are a system of reliable measures of patient–reported health status for physical, mental, and social well–being developed with funding under the National Institutes of Health (NIH) Roadmap for Medical Research Initiative to re-engineer the clinical research enterprise (http://www.nihroadmap.nih.gov). PROMIS measures can be across a wide variety of chronic diseases and conditions and in the general population (Cella et al, 2010). One of the PROMIS instruments is a Sexual Functioning Inventory (Jeffery et al, 2009). The PROMIS Sexual Function Inventory provides scores on seven different sub-domains of sexual function: interest in sexual activity, vaginal discomfort (women only), lubrication (women only), erectile function (men only), orgasm, and global satisfaction with sex life. Each question asks respondents to report on their experiences over the past 30 days. With the exception of the orgasm sub-domain, all sub-domain scores are expressed as T scores (mean = 50, standard deviation = 10). While the instrument is intended for broad use, almost all of the development work was with patients with cancer. Research is ongoing to expand development beyond cancer. In testing with 819 individuals (388 males, 430 females, 1 person did not specify sex), correlations between the PROMIS Sexual Functioning Profile and corresponding sub-domains of two well-established measures, the Female Sexual Function Index (FSFI) and the International Index of Erectile Function (IIEF), ranged between .48 and .92. The sub-domains of the instrument discriminate between people who had and had not asked a provider about sexual problems. Test-retest correlations over one month are >.65 for all sub- domains (Sexual Function and Satisfaction Measures User Manual, 2012).
35. Fagerstrom Test for Nicotine Dependence (FTND)/Fagerstrom Test for Nicotine Dependence – Smokeless Tobacco (FTND-ST): The FTND and FTND-ST are both 6-iem self-report measures that assesses severity of nicotine dependence and smokeless tobacco use, subsequently. Questions on both measures probe quantity of nicotine use and pattern.
36. (PTHA-specific) Credibility and Expectancy Questionnaire (CEQ). The CEQ is a 6-item measure that was designed to assess treatment expectancy and rationale credibility for use in clinical outcomes studies (Devilly & Borkovec, 2000). It has been expanded from a 5-item measure designed primarily to assess credibility (Borkovec & Nau, 1972), 4-items of which have been used by both Foa and Resick (P.A. Resick, personal communication, February 22, 2010; E.A. Hembree, personal communication, February 23, 2010; E. B. Foa, personal communication, February 25, 2010) with the name Expectancy of Therapeutic Outcomes (ETO). The 6-item CEQ assesses both whether the person cognitively understands how the therapy works (credibility) as well as whether the person affectively believes that the therapy will work for them personally (expectancy). The 6-item CEQ has been tested in 217 individuals including 68 male Vietnam veterans and 58 female spouses, 69 individuals diagnosed with general anxiety disorder who had received treatment, and 22 individuals who had received either Cognitive Based Therapy (CBT) or Eye Movement Desensitization and Reprocessing (EMDR) for the treatment of PTSD. The scale demonstrated high internal consistency (alpha coefficients ranged from 0.84 to 0.85). Test-retest reliability over a one-week period was found to be 0.82 for expectancy and 0.75 for credibility. The CEQ was able to differentiate between two treatment rationales in one study, one with and one without an encompassing theory while maintaining equivalence between three rationales in another study. Responses to four questions are scored using a 9-point Likert scale (1= not at all, 9= extremely). Responses to two of the questions are scored using an 11-point Likert Scale (0% to 100%). The combined responses are used to generate a score for credibility and another score for expectancy.

37. The Community Reintegration of Injured Service Members (CRIS): The CRIS is a validated instrument using OIF/OEF cohorts and consists of three scales measuring extent of, perceived limitations in, and satisfaction with community reintegration. This is a pen-and-paper, 9-item measure.
38. Tinnitus Functional Index: The Tinnitus Functional Index (TFI) was reported by Henry et al (2014). They report five stages of TFI development through which prototypes were developed, tested, and revised over a four-year period. The TFI has eight subscales that address the intrusiveness of tinnitus, the sense of control the patient has, cognitive interference, sleep disturbance, auditory issues, relaxation issues, quality of life, and emotional distress.
39. PTHA Study Missing Data Assessment: The PTHA Study Missing Data Assessment is a 5-item measure of global symptom improvement for headache and PTSD, perception of satisfaction and burdensomeness of study treatments, and reason(s) for discontinuing study participation. This assessment will be used to assess missing data randomness and to categorize participants who discontinued study participation as a way to help impute missing data and improve future studies with these treatments in similar populations.
