# Supplementary material for: Cognitive Behavioral Therapy for Veterans With Comorbid Posttraumatic Headache and Posttraumatic Stress Disorder Symptoms: A Randomized Clinical Trial
Source: JAMA Neurol. 2022 Jun 27;79(8):746–57. doi: 10.1001/jamaneurol.2022.1567 (PMC9237802; doi:10.1001/jamaneurol.2022.1567)
Supplement: Supplement 2. — Statistical Analysis Plan [file jamaneurol-e221567-s002.pdf]

Appendix

Statistical Analysis Plan

Randomized Clinical Trial of Cognitive-Behavior Therapy for Posttraumatic Headache

NCT02419131

Principal Investigator: Donald D. McGeary, PhD, ABPP
University of Texas – Health Science Center at San Antonio

Statistical Analysis Plan (SAP) Version: 1.0: August 5, 2019

Senior Biostatistician: Timothy Houle, PhD
Massachusetts General Hospital, Harvard Medical School

Biostatistician: David E. Reed, II, PhD
University of Texas – Health Science Center at San Antonio

Introduction

Background and rationale

Posttraumatic headache (PTHA) is a singularly unique and vexing by-product of traumatic brain injury (TBI) offering numerous challenges both to the individuals experiencing them as well as the medical providers tasked with treating this growing problem (Monteith et al., 2009). Although PTHA is not a new phenomenon (there are reports of posttraumatic headache dating back to the 1700s), very little is known about headaches with onset or exacerbation after TBI.

Despite the significant incidence of PTHA among individuals who suffer TBI, very little is known about its treatment. To date, there are few controlled studies of PTHA treatment, and the nesting of PTHA symptoms within postconcussion syndrome makes this population unresponsive to typical primary headache medications. Many PTHA sufferers attempt self-treatment through the use of acetaminophen, ibuprofen, and opiate/triptan medication (usually prescribed for other reasons). Unfortunately, these over-the-counter or self-prescribed medications do not effectively address PTHA symptoms and may result in headache worsening through medication overuse. Over one-third of PTHA patients are referred to Neurology, where they are typically prescribed anticonvulsants, tricyclics, or gabapentin. Patients with PTHA referred to Neurology usually present with more severe and persistent PTHA symptoms, and few benefit from Neurology intervention. Less than one-third of those referred to Neurology actually present for their first appointment, and 40% of those who attend a first appointment fail to return for subsequent appointments. As a result, there are few studies of medication treatment in PTHA, and many patients refuse medication interventions due to concerns about headache worsening with medication overuse, inability to complete follow-up due to mTBI and PTHA disability, dislike of medications and side effects, and general mistrust of the medical system. Based on significant concerns about medical treatments and poor response to medical interventions, it is strongly recommended that PTHA be addressed through a comprehensive multidisciplinary treatment program offering evidence-based, non-medical interventions for headache management.

Psychiatric comorbidities are common in headache (Breslau et al., 2003), and comorbid psychopathology has been linked to alterations in headache evolution and treatment response (Antonaci et al., 2011). Ruff et al. (2012) estimate that more than 80% of Veterans diagnosed with mTBI and PTHA also have PTSD. Although comorbid headache and PTSD may alter the effectiveness of interventions, there is some evidence to suggest that addressing PTSD directly can ameliorate health symptoms like headache (Shipherd et al., 2013). There are no studies, however, examining the influence of PTSD comorbidity in posttraumatic headache in a military population, so the gaps in PTHA intervention extend beyond targeted non- medication interventions to PTSD treatments that address PTHA through alterations in PTSD symptom severity. In fact, an ongoing PTSD trial overseen by Dr. Patricia Resick, has revealed subjective reports of headache improvement among military service members who are successfully treated for PTSD. We have chosen to add a treatment arm to this study design in which participants with PTHA and comorbid PTSD or PTS symptoms will be treated with a gold-standard, manualized PTSD intervention (Cognitive Processing Therapy; CPT). Both CPT and the manualized headache intervention rely upon cognitive behavioral mechanisms that overlap and may produce relief for both disorders.

Objectives

Primary Objectives

The primary aim of the study is to compare two talk therapies, an eight session CBT intervention for headaches (CBTH) and a 12 session Cognitive Processing Therapy – Cognitive Only (CPT), to Treatment as Usual (TAU) in their ability to decrease headache disability scores (HIT-6) and post-traumatic stress scores (PCL-5) at a clinically meaningful level.

Headache Disability

Hypothesis 1. The CBTH, CPT, and TAU interventions differ in a statistically significant degree in headache disability at posttreatment, measured by the HIT-6. Where $\mu_{CBTH,}$ μ_{CPT} and μ_{TAU} represent the adjusted mean HIT-6 scores at posttreatment, adjusted by HIT-6 scores measured at baseline.

$$H_0: \mu_{CBTH} = \mu_{CPT} = \mu_{TAU}$$

$$H_1: \mu_{CBTH} \neq \mu_{CPT} \neq \mu_{TAU}$$

Only if the null hypothesis is rejected, two planned comparisons will be conducted among selected arms:

Hypothesis 1a. The CBTH intervention, compared to TAU, results in a statistically significant between groups change (decrease) in headache disability at posttreatment, measured by the HIT-6.

$$H_0: \mu_{CBTH} = \mu_{TAU}$$

$$H_1: \mu_{CBTH} \neq \mu_{TAU}$$

Hypothesis 1b. The CPT intervention, compared to TAU, results in a statistically significant between groups change (decrease) in headache disability at posttreatment, measured by the HIT-6.

$$H_0: \mu_{CPT} = \mu_{TAU}$$

$$H_1: \mu_{CPT} \neq \mu_{TAU}$$

PTSD Symptoms

Hypothesis 2. The CBTH, CPT, and TAU interventions differ in a statistically significant degree in post-traumatic stress at posttreatment, measured by the PCL-5. Where $\mu_{CBTH,}$ $\mu_{CPT,}$ and μ_{TAU} represent the adjusted mean PCL-5 scores at posttreatment, adjusted by PCL-5 scores measured at baseline.

$$H_0: \mu_{CBTH} = \mu_{CPT} = \mu_{TAU}$$

$$H_1: \mu_{CBTH} \neq \mu_{CPT} \neq \mu_{TAU}$$

Only if the null hypothesis is rejected, two planned comparisons will be conducted among selected arms:

Hypothesis 2a. The CBTH intervention, compared to TAU, results in a statistically significant between groups change (decrease) in PTSD symptoms at posttreatment, measured by the PTSD checklist-5.

$$H_0: \mu_{CBTH} = \mu_{TAU}$$

$$H_1: \mu_{CBTH} \neq \mu_{TAU}$$

Hypothesis 2b. The CPT intervention, compared to TAU, results in a statistically significant between groups change (decrease) in PTSD symptoms at posttreatment, measured by the PCL-5.

$$H_0: \mu_{CPT} = \mu_{TAU}$$

$$H_1: \mu_{CPT} \neq \mu_{TAU}$$

Secondary Objectives

There are two secondary objectives. The first objective is to determine whether the CBTH group, compared to the CPT group, results in a greater decrease in HIT-6 and PTSD scores. The second objective is to assess between group differences related to longitudinal change in average headache frequency, reported twice daily over a 14-day period as a secondary outcome. Participants will be asked to report the number of headaches they had experienced since the last reporting period.

<u>*Comparison Between CBTH and CPT*</u>

As secondary analyses, the CBTH intervention will be directly compared to the CPT intervention on the primary outcomes.

Hypothesis 3a. The CBTH intervention, compared to CPT results in a statistically significant between groups change (decrease) in headache disability at posttreatment, measured by the HIT-6. Where μ_{CBTH} and μ_{CPT} represent the adjusted mean HIT-6 scores at posttreatment adjusted by HIT-6 scores measured at baseline.

$$H_0: \mu_{CBTH} = \mu_{CPT}$$

$$H_1: \mu_{CBTH} \neq \mu_{CPT}$$

Hypothesis 3b. The CBTH intervention, compared to CPT, results in a statistically significant between groups change (decrease) in PTSD symptoms at posttreatment, measured by the PCL-5. Where μ_{CBTH} and μ_{CPT} represent the adjusted mean PCL-5 scores at posttreatment adjusted by PCL-5 scores measured at baseline.

$$H_0: \mu_{CBTH} = \mu_{CPT}$$

$$H_1: \mu_{CBTH} \neq \mu_{CPT}$$

<u>*Headache Frequency*</u>

Hypothesis 4. The CBTH, CPT, and TAU interventions differ in a statistically significant degree in average headache frequency at posttreatment. Where $\mu_{CBTH,}$ μ_{CPT} and μ_{TAU} represent the adjusted average headache frequency at posttreatment, adjusted by average headache frequency measured at baseline.

$$H_0: \mu_{CBTH} = \mu_{CPT} = \mu_{TAU}$$

$$H_1: \mu_{CBTH} \neq \mu_{CPT} \neq \mu_{TAU}$$

Hypothesis 4a. The CBTH intervention, compared to TAU, results in a statistically significant between groups change (decrease) in average headache frequency at posttreatment.

$$H_0: \mu_{CBTH} = \mu_{TAU}$$

$$H_1: \mu_{CBTH} \neq \mu_{TAU}$$

Hypothesis 4b. The CPTC-C intervention, compared to TAU, results in a statistically significant between groups change (decrease) in average headache frequency at posttreatment.

$$H_0: \mu_{CPT} = \mu_{TAU}$$

$$H_1: \mu_{CPT} \neq \mu_{TAU}$$

Hypothesis 4c. The CBTH intervention, compared to CPT, results in a statistically significant between groups change (decrease) in average headache frequency at posttreatment.

$$H_0: \mu_{CBTH} = \mu_{CPT}$$

$$H_1: \mu_{CBTH} \neq \mu_{CPT}$$

<u>*Preventive Medication Use*</u>

Hypothesis 4. The CBTH, CPT, and TAU interventions differ in a statistically significant degree in frequency of preventive medication use for headache at posttreatment. Where $\mu_{CBTH,}$ μ_{CPT} and μ_{TAU} represent the adjusted average preventive medication use at posttreatment, adjusted by average preventive medication use measured at baseline.

$$H_0: \mu_{CBTH} = \mu_{CPT} = \mu_{TAU}$$

$$H_1: \mu_{CBTH} \neq \mu_{CPT} \neq \mu_{TAU}$$

Hypothesis 4a. The CBTH intervention, compared to TAU, results in a statistically significant between groups change (decrease) in preventive medication use at posttreatment.

$$H_0: \mu_{CBTH} = \mu_{TAU}$$

$$H_1: \mu_{CBTH} \neq \mu_{TAU}$$

Hypothesis 4b. The CPTC-C intervention, compared to TAU, results in a statistically significant between groups change (decrease) in preventive medication use at posttreatment.

$$H_0: \mu_{CPT} = \mu_{TAU}$$

$$H_1: \mu_{CPT} \neq \mu_{TAU}$$

Hypothesis 4c. The CBTH intervention, compared to CPT, results in a statistically significant between groups change (decrease) in preventive medication use at posttreatment.

$$H_0: \mu_{CBTH} = \mu_{CPT}$$

$$H_1: \mu_{CBTH} \neq \mu_{CPT}$$

Trial Design

Overall Design

This research is designed as a three-parallel-arm randomized clinical trial comparing an 8-session manualized CBT intervention for headaches (CBTH) to the 12 session Cognitive Processing Therapy – Cognitive Only (CPT) to Treatment as Usual (TAU) for patients with co-morbid symptoms of PTHA and posttraumatic stress following military deployment and combat trauma. Clinic-based assessments will occur prior to treatment and 1-, 3-, and 6-months (following treatment completion). Blood will be collected prior to treatment, during two of the sessions of the 6-week treatment phase for all participants and at 1- and 6-months following treatment in collaboration with the Consortium to Alleviate PTSD (CAP) Genomics and Basic Science Core to examine gene expression profiles links with PTHA that may be predictive of treatment outcomes. Veterans randomized to the TAU group will have an option to be treated clinically with either CBTH or CPT after their 6-month follow-up assessment is complete.

Population

Inclusion Criteria

Participants in the study will be adult (aged 18 and above) United States military Veterans and active duty personnel with military service during a post-9/11 deployment. They must have sustained a traumatic head injury and have been diagnosed or reported symptoms consistent with chronic (> 3 months) posttraumatic headache attributed to a traumatic injury. We plan to enroll chronic PTHA only due to the very low likelihood of headache remission after 3 months, the disability associated with chronic PTHA, and the high prevalence of chronic versus acute PTHA in this population. A positive PTHA diagnosis was indicated for individuals with de novo headache onset after a concussion or exacerbation of pre-existing headache symptoms (i.e., increased frequency, duration, or intensity), which is consistent with the existing International Headache Classification of Headache Disorders – Third Edition (ICHD-3) diagnostic criteria for PTHA with the exception of onset latency between headache (or headache worsening) of up to one month instead of seven days. The ICHD-3 criteria for PTHA clearly state that the 7-day onset latency criterion has no foundation in extant data and is "somewhat arbitrary" (IHS, 2018). The likelihood of de novo headache up to 1 month after a head injury is very low (Evans, 2008), and there is concern that limiting diagnosis to the arbitrary 7-day onset criterion may unnecessarily exclude some veterans who present with posttraumatic headache (Theeler & Erickson, 2012). Thus, PTHA inclusion for this trial will be based on collaborative diagnosis between the PI, at least one PRC/Polytrauma System of Care (PSC) co-PIs and other team headache experts if symptoms are consistent with chronic PTHA but onset latency was beyond 7 days (the team must come to a consensus on these inclusion). If a diagnosis of PTHA is already documented in the participant's medical record (e.g., CPRS) then they will be included in the trial. If taking headache medication, participants must have agreed to work with their prescriber to remain on stable doses of any prescribed headache medications for the duration of the intervention and through the follow-ups as much as possible and as medically indicated. Participants must have also reported on the Clinician Administered PTSD Scale (CAPS-5) an exposure to a traumatic event (Criterion A) and at least one intrusion symptom (Criterion B). There is some evidence suggesting 40% comorbidity between PTSD and new onset headache, so it is reasonable to assume that at least half of all PTHA participants recruited for this study will have PTHA and comorbid PTS symptoms. The inclusion of PTS symptoms in this sample was vital based on reports indicating that PTS symptoms and PTSD actually increase vulnerability to PTHA and chronic headache in military populations.

Exclusion Criteria

Participants who had a recent and significant change in the nature of headache symptoms over the last 6 weeks prior to screening (as determined by the investigators, confirmed by patient report, and/or documented in the medical record) are excluded from the study. Other excluded participants include those currently enrolled in CPT or prolonged exposure treatment for PTSD (also based on patient report or documentation in the medical record). We will exclude veterans who meet criteria for medication overuse for headache based on the Structured Diagnostic Headache Interview- Revised (Brief Version; SDIH-R) and clinical judgment. Excluded veterans also include those unable to read or speak English at a 6th grade level, those who have a psychiatric hospitalization in the last 6 months before initial assessment, are pregnant or plan to become pregnant during the trial (due to concerns about pregnancy-induced headache that may obscure findings), demonstrate significant cognitive impairment that could impact treatment adherence/benefit, and those who have a present psychiatric problem that warrants immediate treatment as indicated by the computerized patient record system (CPRS), flagged by an independent investigator during evaluation, or confirmed by a clinician through screening or review of CPRS notes.

Primary Outcomes

Two co-primary outcomes are defined that measure headache disability and PTSD symptoms.

Headache disability (HIT-6): The HIT-6 is a 6-item assessment that measures headache-related interference and disability. Participants answer the items on a 5-point Likert scale ranging from "Never" to "Always." Sums are used as scale scores, and items are coded as follows: "Never" = 6; "Rarely" = 8; "Sometimes" = 10; "Very Often" = 11; and "Always" = 13.

Posttraumatic stress disorder symptoms (PCL-5) - The PCL-5 is a self-report 20-item assessment measuring each symptom of PTSD as defined by the DSM-5 on a 5-point Likert scale: "Not at All" = 0; "A little bit" = 1; "Moderately" = 2; "Quite a bit" = 3; "Extremely" = 4. Items are summed for a total scale score. Both the HIT-6 and PCL-5 are assessed at each time point (pretreatment, posttreatment, 3-month follow-up, and 6-month follow-up).

Secondary Outcomes

Because this trial will be conducted under the auspices of the Consortium to Alleviate PTSD (CAP), a well-validated, standardized battery of secondary assessments will be administered as CAP common data elements. See Appendix for a comprehensive list of the domains and measures.

Patient Demographics

Patient characteristics will be evaluated that clearly define the population being examined, including demographic information (e.g., age, gender, race/ethnicity, education, employment and household income). No information will be reported or displayed that allow identification of the individual participants.

Ancillary Patient Characteristics

Patient characteristics unrelated to basic demographic information that help define the population will also be evaluated. Because this is a veteran population, information pertaining to one's military service (e.g., military service branch, years in the service, and number of deployments) will be presented. Mental and physical health information, such as medication use, diagnoses, comorbid symptoms of anxiety, depression, and problems with sleep will also be presented. Depending on variable distributions, means, standard deviations, frequencies, and/or range of values will be presented. Additionally, treatment group imbalances will be evaluated using absolute standard differences (ASD).

Sample Size

A recent study helped to guide a determination of what constitutes a clinically meaningful change in perceived headache disability and the proposed study is powered in light of these insights. In the primary care setting, headache impact test (HIT-6) changes of 2.5 points (95% CI: -3.3 to -1.7) were described as "somewhat better" and changes of 5.9 (95% CI: - 7.6 to -4.1) were described as "much better." We considered a meaningful change as somewhere between these two global impressions and used this marker as an index of clinically significant between-group differences. Our study is powered to detect an effect size of 2.8 points between groups after controlling for baseline scores (i.e., a residualized change) and constitutes an effect that would be meaningful to most headache sufferers. Because there are two primary outcomes, alpha level was set at $\alpha < .025$ to prevent Type I error (see Type-I error strategy, below).

Assuming an alpha level of 0.025, group sample sizes of n = 64 (N = 192), and a moderate correlation between the baseline scores and final endpoints (r = 0.50), we will have power = 0.80 to detect an effect size of d = 0.52 between both of the joint primary comparisons. In realistic terms, this will allow us to detect a change of 2.8 points on the HIT-6 between the active treatments and the control. Changes smaller than this magnitude are unlikely to be clinically meaningful.

Interim Analyses

There are no interim analyses planned. Confidential review by a Data Safety Monitoring Board (DSMB) will occur routinely, and we will not apply stopping rules for futility or superiority.

Ending the Trial

The trial will cease when targeted enrollment is obtained.

Analysis Populations

Planned analyses will include both intention to treat (ITT) and per protocol (PP) population sets. The primary analysis will be conducted on a modified ITT set defined as randomized individuals who actually receive one or more treatment sessions. The PP set will be restricted to those individuals who actually complete 75% or more treatment sessions (6 sessions of CBTH and 9 sessions of CPT) and complete the relevant assessment occasions. The primary and secondary analyses will be conducted on both the ITT and PP sets and sensitivity analyses will estimate differences in primary and secondary outcomes conditional on the population.

Analyses

Statistical Assumptions

Statistical assumptions of each model will be considered, including distributions and variance of outcome variables. Means, standard deviations, frequencies, histograms, and levels of skewness and kurtosis will be considered in determining how best to represent variables of interest. If these assumptions are violated, which may be assessed using prescribed tests for such violations, proper adjustments to the model will be considered. Also considered will be the particular nature of each variable of interest as it relates to the specific subject matter of the variable, and whether this variable should be represented best by considering it a nominal, ordinal, interval, or ratio. For instance, if participants endorse either many or very few depression symptoms, a binomial distribution (i.e., logistic regression) may be specified indicating possible depression diagnosis as yes/no. This would be consistent with a clinical perspective of depression.

Missing Data

We have developed a plan, as part of this protocol, to contact participants who dropout from treatment or are lost to follow-up to assess for reasons for data missingness and status of PTHA and PTSD symptoms at the time of dropout contact. We will use data from this data recovery protocol to guide Multiple imputation (MI) of missing data that will be used to obtain an intention to treat (ITT) philosophy and the multiply imputed set will serve as the basis for the primary analysis. Missing data will be imputed multiple times (m = 5), conditional on baseline participant characteristics, headache characteristics, and previous measures of the outcome variables. The combined results will be utilized in the final model, including all individuals who were allocated to a treatment arm according to the ITT definition. The treatment effect estimates from the MI procedure will be compared to single imputation and last observation carry forward to ensure robustness (Wright & Sim, 2003). Variables that are time variant (e.g., brief-inventory of psychosocial functioning) and invariant (e.g., number of deployments) will be examined as to whether they differentially predict missingness and to assess whether the missing at random assumption has been met (Enders, 2010). If the assumption has not been met, variables that contribute to that violation will be included in the final sensitivity analysis.

Type-I error Strategy

To account for the co-primary outcomes while maintaining an acceptable type-I error rate, each outcome will be assigned an $\alpha = 0.025$ ($\alpha = 0.05 / 2 = 0.025$). Additionally, because there are three treatment arms, an omnibus test result will serve as a necessary gatekeeper requirement before conducting two planned contrasts within each outcome. For these contrasts, each will then be interpreted at the $p < 0.025$ level of statistical significance. No multiplicity adjustments will be made for secondary outcomes.

Primary Analyses

Generalized linear models (GLM) will be used to examine both of the primary outcomes. Specifically, a GLM model will be conducted examining the superiority of CBTH and CPT in alleviating symptoms of headache disability (HIT-6) and PTSD symptoms (PCL-5) compared to TAU at posttreatment. Two separate models will be conducted (one for each outcome) whereby baseline headache disability scores or PTSD symptoms (depending on which outcome is being examined) are entered with a fixed-effect treatment group variable (i.e., CBTH, CPT, TAU) specified as predictors. This is akin to an ANCOVA approach (Vickers, 2001; Raudenbush & Bryk, 2001) but allows for specification of the outcome distribution using an appropriate distribution and link function. It is anticipated that a normal distribution with log link will fit the data well, but a log-link will be examined for improved model fit, and utilized in the primary models, if necessary.

Only if the omnibus test of group is statistically significant at the adjusted level of statistical significance (see below), planned post-hoc contrasts will be examined to determine whether Hypotheses 1a (HIT-6 scores: CBTH v. TAU) 1b (HIT-6 scores: CPT v. TAU), 2a (PCL-5: CBTH v. TAU), and 2b (PCL-5: CPT v. TAU) are supported.

Secondary Analyses

Many secondary analyses are planned and each will be conducted using Generalized linear models (GLM). GLM is a flexible analytic tool as it allows for the specification of both categorical and continuous independent and dependent variables. It also allows for the specification of outcome variable distributions (e.g., normal, binomial) and link functions (e.g., identity and log) in case of non-normal data.

Similar to the primary analysis, an ANCOVA approach (Vickers, 2001; Raudenbush & Bryk, 2001) utilizing GLM will be specified for each secondary objective. HIT-6 scores and PTSD symptom scores at posttreatment will be assessed to determine whether CBTH is superior to CPT in two separate GLMs (Hypotheses 3a and 3b, respectively). Change in average headache frequency will also be assessed utilizing GLMs, determining whether CBTH is superior to TAU (Hypothesis 4a), whether CPT is superior to TAU (Hypothesis 4b), and whether CBTH is superior to CPT (Hypothesis 4c) in reducing average headache frequency. The model will control for baseline scores of HIT-6, PTSD symptoms, and average headache frequency (depending on which secondary outcome is being assessed) and include a fixed treatment group variable.

Post-hoc contrasts will be examined to determine whether Hypothesis 3a (HIT-6: CBTH v. CPT), Hypothesis 3b (PCL-5: CBTH v. CPT), Hypothesis 4a (average headache frequency: CBTH v. TAU), Hypothesis 4b, (average headache frequency: CPT v. TAU), and Hypothesis 4c (average headache frequency: CBTH v. CPT) are supported.

Additional secondary analyses will involve examining changes in the secondary outcomes specified in the appendix. These outcomes will also be evaluated using GLM and examine treatment effects as fixed factors.

The pattern of within-person change in the daily headache diaries will be evaluated using polynomial linear mixed-effects models with random intercepts for participants to account for repeated assessments. Time will be modeled using a suitable polynomial (e.g., quadratic) and treatment group fixed effects will be examined using an interaction with time (e.g., treatment group x time). This will allow the evaluation of differential change in the daily slopes as a function of group assignment.

Sensitivity Considerations

Several sensitivity models are planned to evaluate the estimates of treatment effect under the context of differing assumptions.

The primary models will be specified using an additional set of a priori selected set of clinical predictors that might impact treatment effect. Baseline levels of affective state, sleep, age, headache characteristics, number of lifetime head injuries and sex will be specified as fixed covariate predictors, as these variables are known to be associated with headache disability and PTSD and as these variables are known to be associated with headache disability and PTSD. The conditional estimates based on this model will be informally compared to that from the primary model with predictions from this model used to estimate marginal treatment effects for subpopulations of interest.

As above, treatment heterogeneity will be examined using interaction terms between clinical predictors and treatment assignment (e.g., sex x treatment). Because such interaction terms are difficult to power, and subject to spurious associations, they will be treated as exploratory and used in a hypothesis-generating context.

As above, in order to ensure treatment effects are sufficiently robust, baseline variables known to be potentially associated with headache disability or PTSD symptoms will be entered into the models as covariates for key secondary outcomes (specified in the secondary objectives).

As described in the missing data approach, several different missing data models will be specified to evaluate the treatment effects under differing missing value assumptions.

Statistical Software

The latest versions of R and RStudio, SAS, and Mplus will be used to examine all hypotheses and analyze all data.

Consort (2010)

A consort (2010) will be presented illustrating the number of individuals who were screened, were found eligibile for the trial, who withdrew from the study, and any other information that defines how the total number of participants in the intent to treat and per protocol analyses was generated. Included in this will be those who withdrew, why they withdrew, and at what point in the trial they withdrew. Any publications stemming from the analyses will conform to the CONSORT 2010 reporting guidelines.
