# Supplementary material for: Cognitive Behavioral Therapy for Veterans With Comorbid Posttraumatic Headache and Posttraumatic Stress Disorder Symptoms: A Randomized Clinical Trial
Source: JAMA Neurol. 2022 Jun 27;79(8):746–57. doi: 10.1001/jamaneurol.2022.1567 (PMC9237802; doi:10.1001/jamaneurol.2022.1567)
Supplement: Supplement 3. — eTable 1. Treatment Elements eTable 2. ANOVA for the Primary Analyses eTable 3. Treatment × Postintervention Time (1, 3, 6 months) eTable 4. Primary Outcomes and Sensitivity Analyses eTable 5. Descriptive Statistics for Primary and Secondary Outcomes eFigure 1. GAD-7 eFigure 2. Insomnia Severity Index (ISI) eFigure 3. PHQ-9 eFigure 4. Headache Frequency (28-day month) eFigure 5. Headache Intensity (0-10) eTable 6. Secondary Outcome Plots Model Contrasts eTable 7. Multiple Imputation eTable 8. Results of the Pooled Estimates eTable 9. Effect Plot [file jamaneurol-e221567-s003.pdf]

Supplementary Online Content

McGeary DD, Resick PA, Penzien DB, et al. Cognitive behavioral therapy for veterans with comorbid posttraumatic headache and posttraumatic stress disorder symptoms: a randomized clinical trial. *JAMA Neurol*. Published online June 27, 2022. doi:10.1001/jamaneurol.2022.1567

eTable 1. Treatment Elements

eTable 2. ANOVA for the Primary Analyses

eTable 3. Treatment × Postintervention Time (1, 3, 6 months)

eTable 4. Primary Outcomes and Sensitivity Analyses

eTable 5. Descriptive Statistics for Primary and Secondary Outcomes

eFigure 1. GAD-7

eFigure 2. Insomnia Severity Index (ISI)

eFigure 3. PHQ-9

eFigure 4. Headache Frequency (28-day month)

eFigure 5. Headache Intensity (0-10)

eTable 6. Secondary Outcome Plots Model Contrasts

eTable 7. Multiple Imputation

eTable 8. Results of the Pooled Estimates

eTable 9. Effect Plot

This supplementary material has been provided by the authors to give readers additional information about their work.

© 2022 McGeary DD et al. *JAMA Neurology.*

eTable 1. Treatment Elements

A summary of the core treatment elements for each behavioral treatment arm.

CBT for Headache (CBTH)	Cognitive Processing Therapy (CPT)
Introduction/education to CBTH, progressive muscle relaxation for headache, identifying stressful situations	Introduction/Education to CPT and Stuck Points
Practice progressive muscle relaxation, discuss exercise/sleep, dealing with setbacks	Meaning of traumatic event and connections to thoughts and feelings
Learn 4-muscle group progressive muscle relaxation, learn diaphragmatic breathing, review sleep hygiene	Identification of thoughts and feelings
Learn relaxation by recall, identification of stressful situations, reframing problems as goals	Identifying and challenging stuck points
Discuss headache prevention, learn cue-controlled relaxation, introduce problem-solving	Challenging assumptions and conclusions
Hand-warming biofeedback, managing thoughts related to stressful situations	Problem-solving and patterns of problematic thinking
Discuss coping with headaches and use of skills, attention diversion during headache	Patterns problematic thinking, challenging beliefs, and safety issues related to self & others
Review skills, post-treatment planning	Safety stuck points, challenging beliefs and trust issues related to self & others
	Judgment issues related to problems with trust, power/control issues related to self & others
	Connecting power/control issues to self-blame, review ways of giving and taking power, esteem issues related to self & others
	Giving/receiving compliments and engaging in pleasant activities, identify and challenge esteem issues and assumptions, intimacy issues
	Identify intimacy issues, review of course and progress

© 2022 McGeary DD et al. *JAMA Neurology.*

eTable 2. ANOVA for the Primary Analyses

In the Table, below, the ANOVA table for the primary analyses reported in the manuscript is reported.

Outcome	Factor	Numerator DF	Denominator DF	F	p-value*
HIT-6	Baseline Hit6	1	116.0	105.8	< 0.001
	Treatment Arm	2	121.3	5.8	0.004
PCL-5	Baseline PCL-5	1	118.4	49.9	< 0.001
	Treatment Arm	2	116.3	3.9	0.023

ANOVA terms estimated using Satterthwaite's method
*Main effect for treatment arm interpreted at p < 0.025

eTable 3. Treatment × Postintervention Time (1, 3, 6 months)

In the table, below, the ANOVA table for the sensitivity analyses that consider the treatment effects over time (treatment x time interaction) is reported.

Outcome	Factor	Numerator DF	Denominator DF	F	p-value*
HIT-6	Baseline Hit6	1	115.6	104.9	< 0.001
	Treatment Arm	2	121.1	5.9	0.004
	Time	2	185.1	1.2	0.29
	Treatment Arm x Time	4	185.3	0.9	0.47
PCL-5	Baseline PCL5	1	117.9	49.4	< 0.001
	Treatment Arm	2	116.2	3.8	0.024
	Time	2	168.8	1.2	0.30
	Treatment Arm x Time	4	168.9	2.3	0.06

ANOVA terms estimated using Satterthwaite's method
*Main effect for treatment arm interpreted at p < 0.025

© 2022 McGeary DD et al. *JAMA Neurology.*

eTable 4. Primary Outcomes and Sensitivity Analyses

In the table, below, least square means (SE) representing the change from baseline estimated from the primary model are reported conditional on posttreatment measurement occasion and stratified by treatment arm. Additionally, treatment contrasts (95%CI) are reported that compare each active treatment arm to treatment per usual (TPU). Two contrasts are reported, one for the primary (unadjusted) model and a sensitivity model that adjusts the associations for an a priori set of predictors.

The sensitivity model adjusted the treatment effects by variables that were theorized to be related to attrition. A linear mixed-effects model was conducted that regressed each primary outcome on treatment, time, treatment x time, baseline levels of the outcome, GAD7 total score, PHQ9 total score, age, race, years of education, seeking VA disability (no vs yes), receiving disability for PTSD (no vs yes), and seeking disability (no vs yes). A random intercept at the level of individual was specified to account for repeated post-treatment measurements, and the model was estimate using maximum likelihood.

Outcome	Month	Treatment per Usual (TPU) N = 64	Cognitive Processing (CPT) N = 64	Cognitive Behavioral (CBTH) N = 65
**HIT6**				
Change from baseline	Post-treatment	-0.7 (0.7)	-2.5 (0.9)	-3.9 (0.8)
Difference vs. TAU (95%CI)		--	-1.7 (-4.2, 0.8)	-3.1 (-5.3, -0.8)
Sensitivity		--	-1.3 (-3.9, 1.3)	-2.8 (-5.1, -0.4)
Change from baseline	3	-0.3 (0.7)	-0.9 (1.0)	-3.9 (0.8)
Difference vs. TAU (95%CI)		--	-0.5 (-3.1, 2.1)	-3.5 (-5.9, -1.2)
Sensitivity		--	-0.7 (-3.4, 2.0)	-3.5 (-5.9, -1.0)
Change from baseline	6	-0.3 (0.8)	-2.6 (0.9)	-4.6 (0.8)
Difference vs. TAU (95%CI)		--	-2.3 (-4.9, 0.3)	-4.3 (-6.6, -1.9)
Sensitivity		--	-2.7 (-5.3, 0.1)	-4.3 (-6.7, -1.8)
**PCL5**				
Change from baseline	Post-treatment	-6.8 (1.9)	-16.2 (2.6)	-11.2 (2.2)
Difference vs. TAU (95%CI)		--	-9.4 (-16.5, -2.4)	-4.6 (-10.9, 1.7)
Sensitivity		--	-12.3 (-19.4, -5.1)	-5.7 (-12.2, 0.8)
Change from baseline	3	-6.9 (2.0)	-17.5 (2.6)	-16.0 (2.3)
Difference vs. TAU (95%CI)		--	-10.7 (-18.1, -3.3)	-9.3 (-16.0, -2.5)
Sensitivity		--	-14.1 (-21.6, -6.5)	-10.7 (-17.7, -3.8)
Change from baseline	6	-7.7 (2.2)	-13.5 (2.6)	-16.7 (2.3)
Difference vs. TAU (95%CI)		--	-5.8 (-13.3, 1.7)	-9.1 (-16.0, -2.2)
Sensitivity		--	-7.4 (-15.0, 0.2)	-8.5 (-15.6, -1.4)

© 2022 McGeary DD et al. *JAMA Neurology.*

eTable 5. Descriptive Statistics for Primary and Secondary Outcomes

Descriptive statistics, mean (SD), for each primary and secondary out is reported by measurement occasion and stratified by treatment arm.

Measure	Time (Months)	TPU	CPT	CBTH
HIT6	0	65.2 (6.4)	66.1 (5.1)	66.1 (5.4)
	1	64.2 (6.6)	63.3 (6.9)	61.9 (6.3)
	3	64.1 (7.1)	64.8 (7.2)	61.3 (8.0)
	6	64.1 (7.3)	63.3 (7.1)	60.9 (8.8)
PCL5	0	49.0 (13.3)	48.6 (14.6)	47.7 (14.7)
	1	41.6 (17.1)	30.0 (20.9)	36.5 (19.7)
	3	43.0 (16.5)	29.9 (20.0)	30.2 (18.2)
	6	41.0 (16.1)	32.9 (21.0)	30.3 (20.0)
GAD7	0	14.6 (4.5)	15.1 (4.4)	13.9 (4.6)
	1	12.6 (5.5)	10.5 (5.4)	10.5 (6.1)
	3	12.5 (5.2)	10.5 (6.0)	10.0 (5.7)
	6	12.5 (5.3)	11.5 (5.1)	9.6 (5.8)
PHQ9	0	16.2 (4.6)	16.9 (5.0)	16.0 (4.9)
	1	14.0 (5.4)	12.4 (5.2)	10.3 (6.4)
	3	12.9 (5.5)	11.7 (5.6)	10.2 (6.1)
	6	13.2 (5.1)	12.4 (5.8)	10.4 (6.0)
ISI	0	19.4 (6.0)	20.6 (5.2)	18.7 (6.1)
	1	18.6 (6.2)	18.7 (7.7)	16.9 (6.3)
	3	19.3 (6.1)	17.7 (8.6)	15.5 (7.9)
	6	17.7 (6.6)	18.4 (6.7)	17.1 (8.4)

© 2022 McGeary DD et al. *JAMA Neurology.*

Secondary Outcome Plots

Boxplots for each of the secondary outcomes are illustrated below. Each treatment arm is depicted using color, with treatment time (0, 1, 3, 6 months) on the x-axis. The boxes represent 25th and 75th percentiles with the median as a line within the box. Vertical lines represent 95%CI with dots representing observed values outside of this range.

eFigure 1. GAD-7

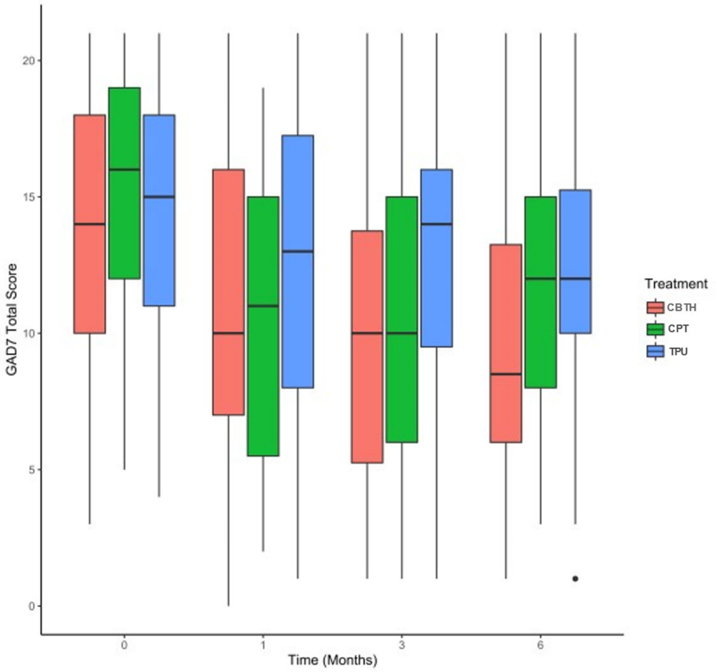

© 2022 McGeary DD et al. *JAMA Neurology.*

eFigure 2. Insomnia Severity Index (ISI)

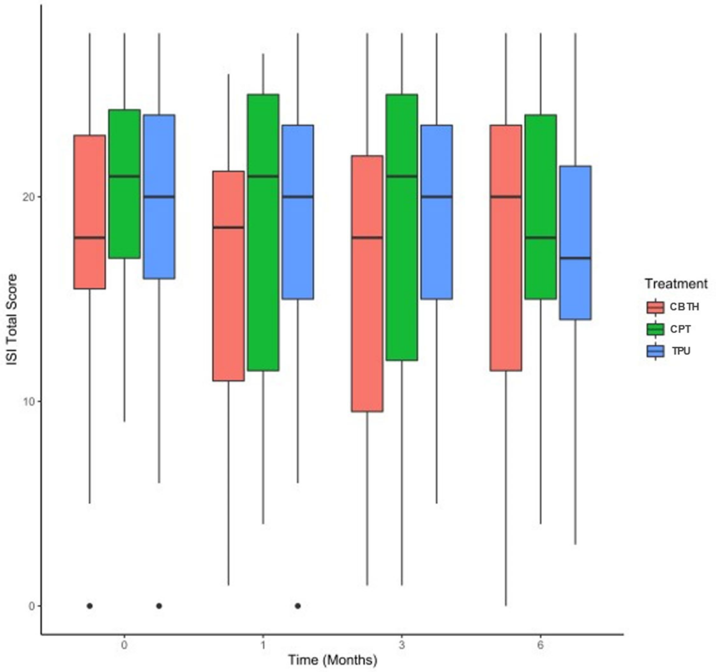

eFigure 3. PHQ-9

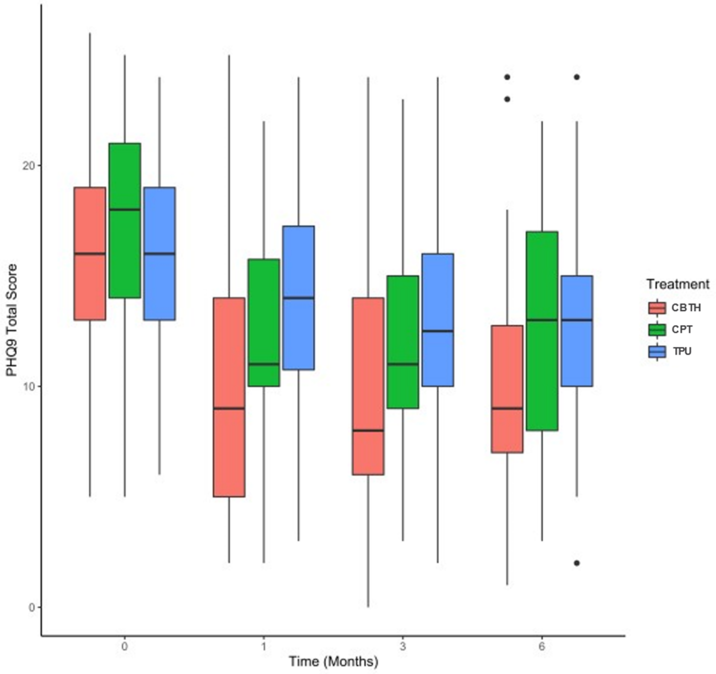

© 2022 McGeary DD et al. *JAMA Neurology.*

eFigure 4. Headache Frequency (28-day month)

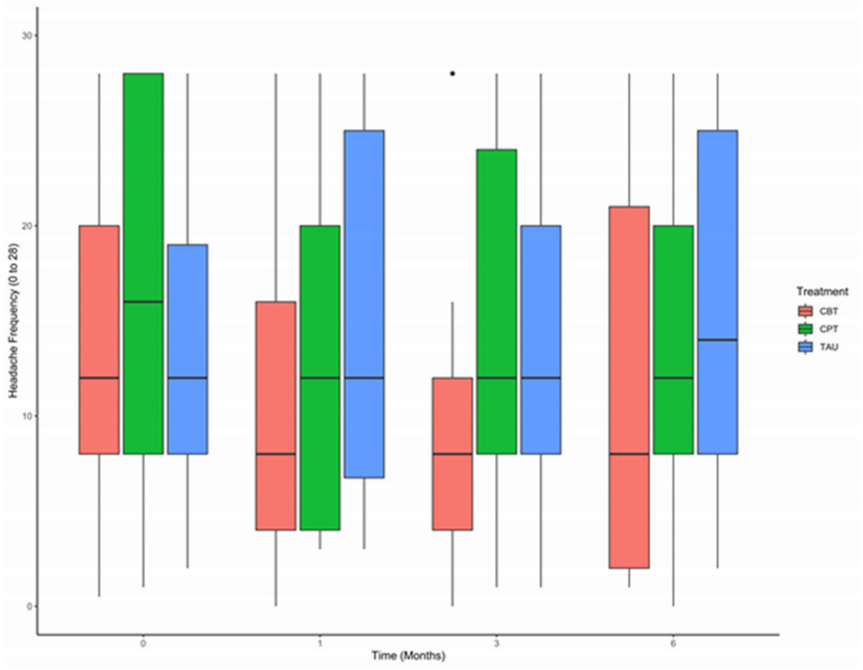

© 2022 McGeary DD et al. *JAMA Neurology.*

eFigure 5. Headache Intensity (0-10)

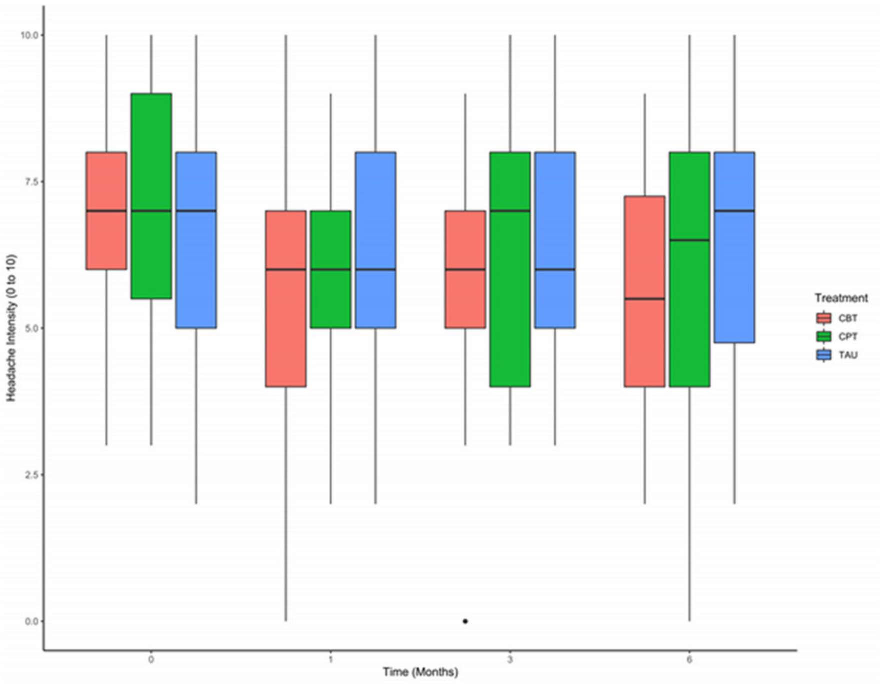

eTable 6. Secondary Outcome Plots Model Contrasts

Each secondary outcome model is conducted with the primary analysis set. The treatment contrast, or Estimate, is the contrast between that treatment arm (CPT, CBTH) and treatment per usual condition (TPU). All models estimate the post-treatment levels of the outcome pooled across measurement occasions (1, 3, and 6 months) conditional on pre-treatment levels of the outcomes and treatment assignment. The models were estimated using linear mixed-effects models that specified a random intercept for participant to account for the repeated measurements for each individual.

**Outcome**	**CPT**			**CBTH**		
	**Estimate**	**95%CI**	**p**	**Estimate**	**95%CI**	**p**
GAD7	-1.8	-3.8 to 0.1	0.07	-1.1	-2.9 to 0.6	0.20
PHQ9	-1.2	-3.1 to 0.7	0.23	-1.7	-3.4 to 0.0	0.05
ISI	-2.0	-4.5 to 0.5	0.12	-0.8	-3.0 to 1.4	0.47
HA- Intensity	-0.3	-1.1 to 0.5	0.47	-0.6	-1.3 to 0.1	0.11
HA-Frequency	-2.1	-5.6 to 1.4	0.26	-2.9	-6.0 to 0.1	0.07

Note: CPT (Cognitive Processing Therapy), CBTH (Cognitive Behavioral Therapy for Headache), HA-Intensity (Headache intensity 0 to 10 Numeric rating scale); HA-Frequency (Headache frequency assuming a 28-day month).

© 2022 McGeary DD et al. *JAMA Neurology.*

eTable 7. Multiple Imputation

Missing data for each outcome, stratified by treatment arm, by each measurement occasion.

	PCL5			HIT6		
Time	TPU<br>N = 64	CBTH<br>N = 65	CPT<br>N = 64	TPU<br>N = 64	CBTH<br>N = 65	CPT<br>N = 64
Baseline	0 (0%)	0 (0%)	0 (0%)	0 (0%)	0 (0%)	0 (0%)
1M	17 (27%)	32 (49%)	41 (64%)	17 (27%)	32 (49%)	41 (64%)
3M	24 (38%)	37 (57%)	43 (67%)	24 (38%)	34 (52%)	43 (67%)
6M	31 (48%)	34 (52%)	42 (66%)	28 (44%)	33 (51%)	41 (64%)

Model: All of the variables that were included in the primary model were included (HIT6, PCL5, Treatment Arm, Time, Baseline HIT6, Baseline PCL5), the baseline (level-2) predictors theorized to be related to missingness (anxiety: GAD7, depression: PHQ9, insomnia: ISI), and several representations of PCL session-level data theorized to be predictive of treatment progress (lowest PCL score during treatment, maximum PCL score during treatment, first PCL score during treatment, and last PCL score during treatment). Please note that no session-level data were recorded for the HIT6 outcome.

In the original statistical analysis plan (SAP), the specified methods for accommodating the intention to treat strategy was to use multiple imputation for any missing data that were observed during any of the post-treatment assessments (post-treatment, 3M, 6M) on either primary outcome (PCL5, HIT6). This strategy specifies subject-level baseline predictors and session-level data to multiply impute the missing outcome measurements. However, during the conduct of the study, it became clear that there would be substantial missing data in these outcome assessments (i.e., > 20% missing values).

In response, the approach to handling missing data was revised to also include a model-based approach that utilized theoretical predictors of missingness and estimated using maximum likelihood to reduce the impact of potential selection bias (see 3.1, above, for a description of this model). This change was made to provide additional estimates of the treatment contrasts in the context of large amounts of missing data, which can pose additional problems for multiple imputation approaches (Madley-Dowd et al., 2019). The maximum-likelihood approaches are reported in Section 3.1 of this supplement. The methods and results for the multiple imputation model are reported here and summarized in the main document.

The multiple imputation model was conducted using different model assumptions. All models were conducted in R 3.4 using packages designed to accommodate the multilevel data structure used in the primary outcome analysis. Specifically, because each participant contributes up to three outcome measurements for each primary outcome, measurement occasions can be viewed as nested within individuals, and can be modeled using either level-1 (time-varying) or level-2 (participant-level) predictors. To accommodate this structure, the 'mitml' (tools for multiple imputation of multilevel models) and 'jomo' packages (joint modeling for multiple imputation of multilevel data) were used (Grund et al., 2016). This set of algorithms assumes a multivariate normal distribution and uses Markov Chain Monte Carlo (MCMC) sampling to sample posterior estimates of imputed values. For this estimation, the default priors were assumed, and m = 100 data sets were imputed using 1000 burn-in iterations and 1000 iterations for each set. In all models, the MCMC chains exhibited good properties including good mixing and suitable effective sample sizes. The primary models were then re-estimated on the imputed data sets and combined using Rubin's rule using the 'testEstimates' function from the 'mitml' package.

© 2022 McGeary DD et al. *JAMA Neurology.*

The extent of missing data is reported in the table.

eTable 8. Results of the Pooled Estimates

	PCL5			HIT6		
Treatment	Pooled Estimate	Pooled 95%CI	p-value	Pooled Estimate	Pooled 95%CI	p-value
CBTH vs TPU	-5.6	-10.6 to -0.6	0.040	-2.4	-4.1 to -0.7	0.006
CPT vs TPU	-6.0	-10.0 to -0.2	0.027	-1.0	-2.8 to 0.8	0.298

Madley-Dowd P, Hughes R, Tilling K, Heron J. The proportion of missing data should not be used to guide decisions on multiple imputation. Journal of clinical epidemiology. 2019 Jun 1;110:63-73.

Grund S, Lüdtke O, Robitzsch A. Multiple imputation of multilevel missing data: An introduction to the R package pan. Sage Open. 2016 Oct;6(4):2158244016668220.

eTable 9. Effect Plot

To estimate the treatment effect, the plan of analysis utilized several different models, each with unique assumptions. In the Figure, below, the estimated contrast between each treatment arm (CBTH, CPT) and treatment per usual (TPU) is displayed for both of the primary outcomes (Hit6, Pcl5). The different approaches resulted in consistent estimates of the treatment effects for the Hit6 and Pcl5 and outcomes. For the plot, the effect estimate is reported using the original scaling of each primary outcome variable.

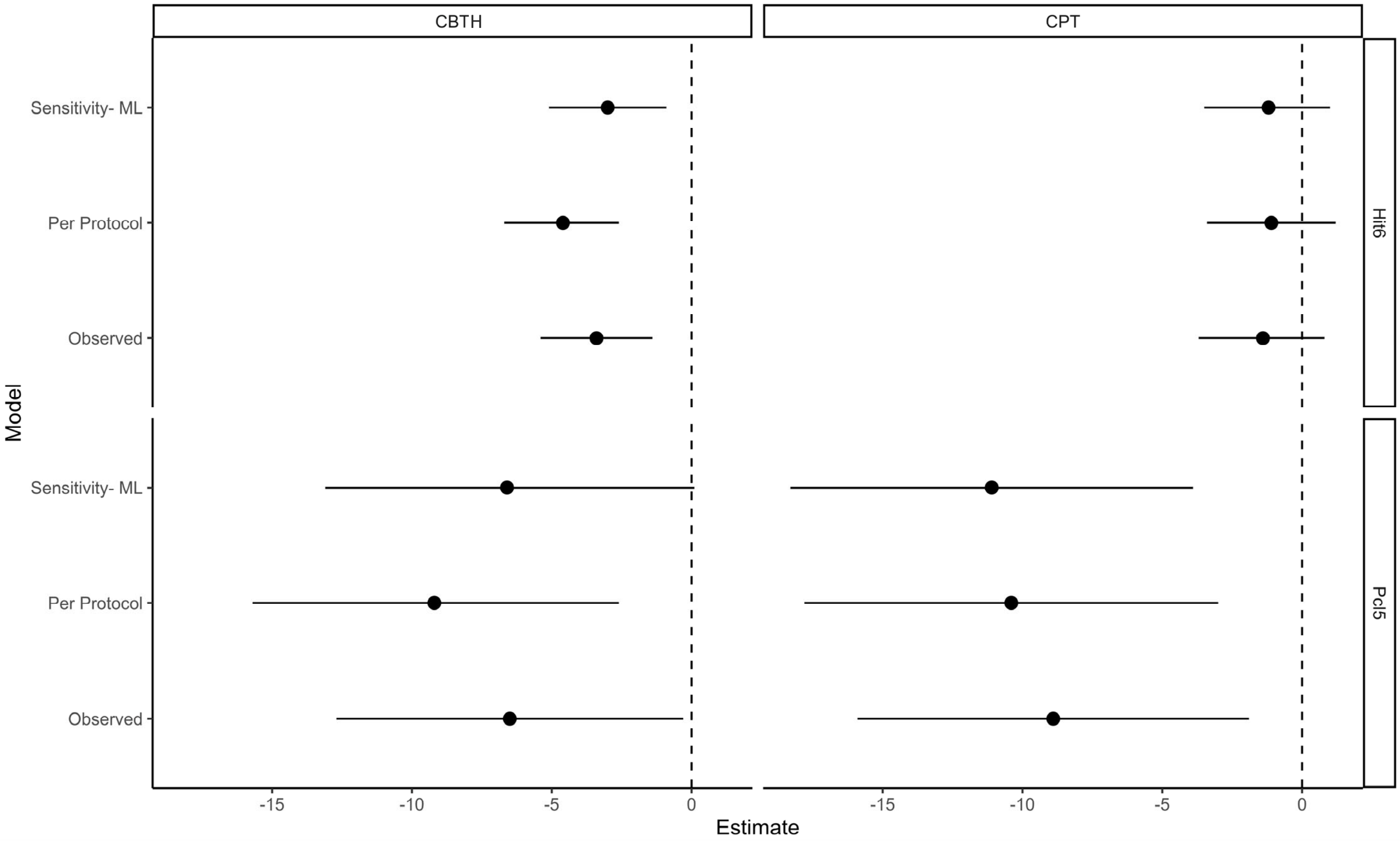

Sensitivity ML: Utilized the primary outcome model (i.e., linear mixed-effects model pooling post-treatment measurements) but included predictors of attrition including baseline levels of both primary outcomes, baseline levels of the Generalized Anxiety Disorder-7 scale (GAD-7) and Patient Health Questionnaire (PHQ-9), age, race, education, VA disability status, PTSD-related disability status, and intention to seek service-connected disability.

Per Protocol: Included only those individuals that met the definition of per protocol based on individual session attendance.

Observed: This is the predefined primary analysis that includes the 6-month interview data that was imputed as the observed score for individuals who dropped-out.

© 2022 McGeary DD et al. *JAMA Neurology.*
