# Supplementary material for: Cognitive Behavioral Therapy for Veterans With Comorbid Posttraumatic Headache and Posttraumatic Stress Disorder Symptoms: A Randomized Clinical Trial
Source: JAMA Neurol. 2022 Jun 27;79(8):746–57. doi: 10.1001/jamaneurol.2022.1567 (PMC9237802; doi:10.1001/jamaneurol.2022.1567)
Supplement: Supplement 4. — Data Sharing Statement [file jamaneurol-e221567-s00.pdf]

Data Sharing Statement

McGeary. Cognitive Behavioral Therapy for Veterans With Comorbid Posttraumatic Headache and Posttraumatic Stress Disorder Symptoms. *JAMA Neurol*. Published June 27, 2022. doi:10.1001/jamaneurol.2022.1567

Data
Data available: Yes
Data types: Deidentified participant data
How to access data: Data requests can be submitted to Don McGeary, PhD at mcgeary@uthscsa.edu
When available: With publication

Supporting Documents
Document types: None

Additional Information
Who can access the data: Data will be made available to researchers whose proposed use of the data has been approved.
Types of analyses: approved secondary and confirmatory analyses.
Mechanisms of data availability: with signed data access agreement
Any additional restrictions: require approval from the STRONG STAR data repository
